# Synthesis and Biopharmaceutical Characterization of Amphiphilic Squalenyl Derivative Based Versatile Drug Delivery Platform

**DOI:** 10.3389/fchem.2020.584242

**Published:** 2020-10-19

**Authors:** Duy-Khiet Ho, Rebekka Christmann, Xabier Murgia, Chiara De Rossi, Sarah Frisch, Marcus Koch, Ulrich F. Schaefer, Brigitta Loretz, Didier Desmaele, Patrick Couvreur, Claus-Michael Lehr

**Affiliations:** ^1^Helmholtz Institute for Pharmaceutical Research Saarland, Helmholtz Centre for Infection Research, Saarbrücken, Germany; ^2^Department of Pharmacy, Saarland University, Saarbrücken, Germany; ^3^INM-Leibniz Institute for New Materials, Saarbrücken, Germany; ^4^Faculté de Pharmacie, Institut Galien Paris Sud, Université Paris-Saclay, Chatenay-Malabry, France

**Keywords:** drug delivery, self-assembly, pegylated, squalenyl derivatives, squalene, nanoparticles, protein-interaction

## Abstract

Limited drug loading capacity (LC), mostly below 5% w/w, is a significant drawback of nanoparticulate drug delivery systems (DDS). Squalenoylation technology, which employs bioconjugation of squalenyl moiety and drug, allows self-assemble of nanoparticles (NPs) in aqueous media with significantly high LC (>30% w/w). The synthesis and particle preparation of squalenoylated prodrugs are, however, not facile for molecules with multiple reactive groups. Taking a different approach, we describe the synthesis of amphiphilic squalenyl derivatives (SqDs) as well as the physicochemical and biopharmaceutical characterizations of their self-assembled NPs as DDSs. The SqDs included in this study are (i) cationic squalenyl diethanolamine (ii) PEGylated SqD (PEG 750 Da), (iii) PEGylated SqD (PEG 3,000 Da), and (iv) anionic squalenyl hydrogen sulfate. All four SqDs self-assemble into NPs in a size range from 100 to 200 nm in an aqueous solution. Furthermore, all NP derivatives demonstrate appropriate biocompatibility and adequate colloidal stability in physiological relevant pH environments. The mucoprotein binding of PEGylated NPs is reduced compared to the charged NPs. Most importantly, this technology allows excellent LC (at maximum of 45% w/w) of a wide range of multifunctional compounds, varying in physicochemical properties and molecular weight. Interestingly, the drug release profile can be tuned by different loading methods. In summary, the SqD-based NPs appear as versatile drug delivery platforms.

## Introduction

Nano-sized drug delivery systems (DDS) having the size range from 10 to 1,000 nm have been investigated intensively to improve the treatment efficacy of severe diseases (Bobo et al., 2016; Ho et al., 2019). DDS protect drugs from biodegradation, improve drug solubility, enhance the delivery of drugs specifically to their target sites and reduce adverse effects (Danhier et al., 2012; Sanna et al., 2012; Kalepu and Nekkanti, 2015; Kim et al., 2017). The major challenge in DDS formulation is to improve the drug loading capacity (LC), which have been mostly reported as lower than 5% w/w while using pharmaceutically accepted excipients (Couvreur, 2013; Ho et al., 2019). With the aim to formulate nanomedicines having a maximized LC, Couvreur et al. (2006) invented the squalenoylation approach. This unique technique creates a prodrug by bioconjugation of a drug molecule and a hydrophobic squalenyl moiety. The squalenoylated prodrug could self-assemble into uniform and stable nanoparticles (NPs) in aqueous solution without using additional surfactants (Desmaële et al., 2012), which cannot be achieved by other lipid prodrugs, e.g., employing stearyl moiety (Couvreur et al., 2006). Importantly, the squalenyl drug bioconjugates, e.g., squalenyl gemcitabine, squalenyl penicillin G, as well as squalenyl dideoxycytidine or didanosine, have not only shown a high LC (>30% w/w) but also improved pharmacological profile and efficacy compared to the parent drugs (Couvreur et al., 2006; Desmaële et al., 2012; Sémiramoth et al., 2012; Hillaireau et al., 2013; Maksimenko et al., 2013; Buchy et al., 2015). Moreover, squalene is a natural lipid, which is also found in humans as a precursor of the cholesterol biosynthesis (Schroepfer, 1981). Hence, using squalenyl derivatives in developing DDS is favorable (Reddy and Couvreur, 2009).

However, it is challenging to bio-conjugate squalene and drug molecules with multiple functional groups or without functional groups (Ralay-Ranaivo et al., 2014). Furthermore, if possible, the chemical synthesis would not be facile and easily scaled-up, and poor solubility of the resulting prodrugs in common solvents could be an additional issue. Taking advantage of squalenoylation approach and extending its use in versatile DDS preparation, Lepeltier et al. (2015) proposed the core-shell structured squalenoylated chitosan NPs allowing the loading of both hydrophilic and hydrophobic compounds. However, the LC in such a system was not as high as expected, and the particles size could not be tuned easily due to the poor solubility of squalenoylated chitosan in common solvents (Lepeltier et al., 2015). Taking a different approach, without employing bioconjugation Ho et al. (2020) reported self-assembled NPs based on an anionic squalenyl derivative (aSq)—squalenyl hydrogen sulfate—which showed excellent simultaneous LC of both hydrophobic alkyl quinolone (~10% w/w) and hydrophilic tobramycin (~30% w/w). Importantly, this DDS also demonstrated an improved penetration through biological barriers, such as biofilms, and enhanced synergistic therapeutic effects of both actives (Ho et al., 2020). Hence, we aimed to expand such a promising DDS platform to further applications, like drug loading of anionic hydrophilic drugs and the minimization of interactions between the NPs and proteins. For these purposes, we here propose, in addition to the synthesis of aSq, the preparation of other squalenyl derivatives (SqDs), namely, (i) cationic squalenyl diethanolamine (cSq), (ii) PEGylated squalenyl derivative (PEG 750 Da, PEG750Sq), and (iii) PEGylated squalenyl derivative (PEG 3,000 Da, PEG3000Sq). Especially, the low molecular weight of SqDs in comparison to excipients used in conventional carrier systems [e.g., from biodegradable polymers like poly (lactic-co-glycolic acid)] promises to lead to a higher LC in these DDS. We studied their ability to self-assemble into NPs in aqueous solution, their morphology, as well as their colloidal stability in biophysically relevant pH milieus and cytotoxicity as surrogate for their biocompatibility. At their first contact with the human body, NPs are mostly confronted with proteins. By adsorption on the NP surface (formation of a “protein-corona”), the proteins can change the characteristics of NPs dramatically (Raesch et al., 2015; Kokkinopoulou et al., 2017). These protein–NP interactions not only play a role for systemic applications but also for non-invasive drug delivery, where the first main challenge is to overcome a protective mucus barrier (e.g., vaginal, pulmonary, gastrointestinal delivery) (Ruge et al., 2013; Murgia et al., 2018). Mucus is a hydrogel consisting mainly of water and mucins, which are high molecular weight glycoproteins with a negative net charge at physiological pH (Bansil and Turner, 2006; Lock et al., 2018; Murgia et al., 2018). One of the main defense mechanisms of mucus against xenobiotics is the physicochemical interaction with mucin glycoproteins (Lai et al., 2007; Lieleg and Ribbeck, 2011; Murgia et al., 2016). In this study, these possible interactions were investigated using nanoparticle tracking analysis (NTA), to evaluate the mucoadhesive and mucoinert characteristics of the charged and PEGylated SqDs, respectively. To assess the LC of SqDs for a broad range of molecules, a representative set of compounds, varying in physiochemical properties and molecular weight (Mw), was chosen. Depending on the drug properties, the loading procedure has been optimized using different methods, namely, (i) coprecipitation, (ii) solvent evaporation, or (iii) dropping. Subsequently, the tuning possibility of drug release by different preparation methods was studied by *in vitro* release profiles of the drug from SqD–NPs, at pH 7.4 (PBS), and 37°C.

## Materials and Methods

### Materials

All chemicals were purchased from Sigma-Aldrich unless otherwise specified. Tetrahydrofuran (HPLC grade) (THF), ethanol, absolute (HPLC grade) (EtOH), ethyl acetate (analytical grade reagent), and Formic Acid Optima LCMS were purchased from Fisher Scientific. Acetonitrile and methanol (MeOH) were obtained from VWR Chemicals. Yeast extract was obtained from Fluka. Bacto™ Tryptone was obtained from BD Biosciences. Luria Bertani (LB) agar was obtained from Carl Roth. Gibco® HBSS (1x) Hanks' Balanced Salt Solution and Gibco® PBS was obtained from Life Technologies. Purified water was prepared by a Milli-Q water purification system (Merck Millipore, Billerica, MA, USA) (called water in the manuscript).

### Synthesis and Characterization of SqDs

**Figure 1:**
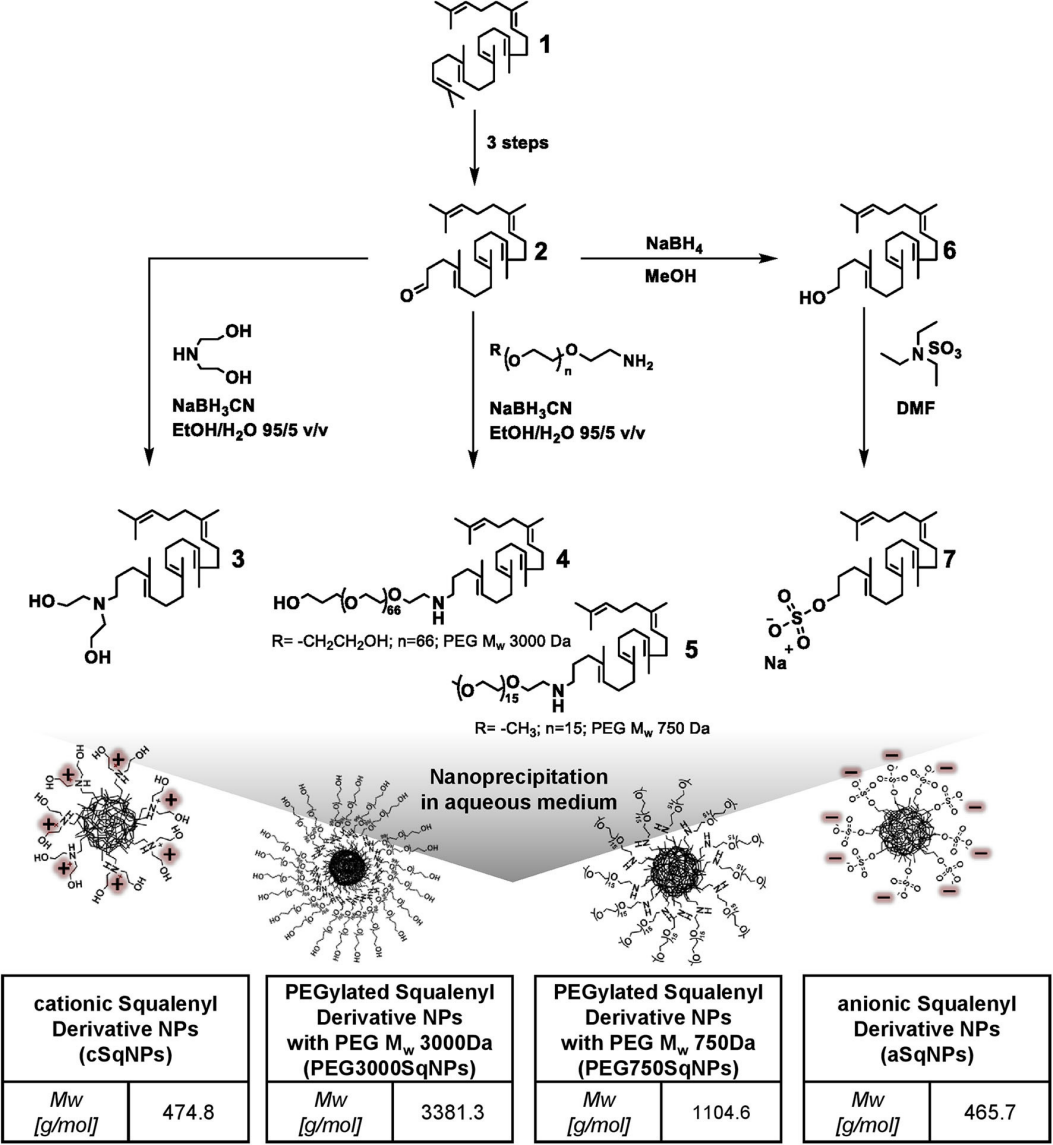
Synthetic scheme of squalenyl derivatives (SqDs) [cationic squalenyl diethanolamine (cSq) (compound 3), PEG3000Sq (compound 4), PEG750Sq (compound 5), squalenyl derivative (aSq) (compound 7)], and schematic sketches of nanoparticles (NPs) upon nanoprecipitation in aqueous solution. The insert tables indicate abbreviation and molecular weight of the corresponding SqD.

The preparation of 1,1′,2-trisnorsqualenic aldehyde (compound 2) and 1,1′,2-trisnorsqualenol (compound 6) (Figure 1) from squalene (compound 1) was performed as reported in previous studies (van Tamelen and Curphey, 1962; Ceruti et al., 1987; Skarbek et al., 2015).

#### Synthesis and Characterization of PEGylated SqDs and cSq

cSq (compound 3), PEG3000Sq (compound 4), and PEG750Sq (compound 5) were synthesized using the same procedure from 1,1′,2-trisnorsqualenic aldehyde (compound 2) (Figure 1). The conjugation was achieved *via* reductive amination reaction between amine and aldehyde groups, using sodium cyanoborohydride (NaBH_3_CN) as reducing agent. Briefly, 1 molar equivalent of 1,1′,2-trisnorsqualenic aldehyde (compound 2) was solubilized in a mixed solvent of EtOH:water (95:5 v/v) at a concentration of 10% w/w. Following the addition of 1.2 molar equivalent of amine—methoxyl-PEG750-NH_2_ (methoxypolyethylene glycol amine, Mw 750 Da), PEG3000-NH_2_ [O-(2-aminoethyl) polyethylene glycol, Mw 3,000 Da], or diethanolamine—the reaction was carried out at room temperature for 3 h. NaBH_3_CN, 1.2 molar equivalent, was subsequently added, and the reaction was kept for an additional 20 h allowing imine reduction. EtOH was then removed under reduced pressure, and water was added, allowing precipitation of the resulted SqD. The product was then collected by centrifugation and washed three times with water. The isolated yield was >90% in all reactions. The cSq product (compound 3) was characterized by ^1^H-NMR (Supplementary Figure 3), ^13^C-NMR (Supplementary Figure 4), and MS (mass spectroscopy), while the PEG750Sq product (compound 5) and PEG3000Sq product (compound 4) were characterized by ^1^H-NMR (Supplementary Figures 5, 7, respectively) and ^13^C-NMR (Supplementary Figures 6, 8, respectively) before further use.

#### Synthesis and Characterization of aSq (Compound 7)

aSq was synthesized from 1,1′,2-trisnorsqualenol as described by Ho et al. (2020) (Figure 1). Briefly, 1 molar equivalent of 1,1′,2-trisnorsqualenol (compound 6) was prepared in DMF (dimethylformamide) at a concentration of 10% w/w. The solution was degassed using N_2_, and 1.1 molar equivalent of TEA. SO_3_ (sulfur trioxide triethylamine complex) was slowly dropped into the solution at room temperature. The temperature was then increased to 60°C. Following 16 h of reaction, the resulting system was quenched with an appropriate amount of water. The solvent mixture, DMF and water, was partly removed under reduced pressure. After being diluted in an excess amount of MeOH, NaOH 1 N was added under stirring. MeOH was subsequently removed under reduced pressure, and water was added allowing precipitation of aSq. The aSq product was extracted using ethyl acetate and dried over MgSO_4_. The solvents were completely removed under reduced pressure at 40°C. The isolated yield was 95%. The aSq product was characterized by ^1^H-NMR (Supplementary Figure 1), ^13^C-NMR (Supplementary Figure 2), and MS (mass spectroscopy) before further use.

### Preparation of Drug-Free SqD–NPs

All drug-free self-assembled SqD–NPs in this study were prepared by nanoprecipitation in aqueous solution as described previously (Fessi et al., 1989; Ho et al., 2020). In brief, a SqD was solubilized in THF and dropped into the water at a speed of 12 drops/min under constant stirring (1,000 rpm). Afterward, THF was evaporated at 40°C, 40 mbar, and 280 rpm using a rotary evaporator (Rotavapor, Büchi, Essen, Germany) resulting in a drug-free SqD–NPs suspension in water, which was further stored at 4°C.

Figure 1 presents the schemes of the optimal representatives of SqD–NPs, including:

- cSq–NPs, which was prepared as follows: 0.15 ml of cSq solution in THF at a concentration of ~7 mg/ml was dropped into 1 ml of water under stirring. THF was then removed under reduced pressure resulting in a cSq–NPs suspension in water at a concentration of 1 mg/ml.
- PEG750Sq–NPs and PEG3000Sq–NPs, which were prepared using the same protocol as follows: 0.5 ml PEGylated SqD in THF:water (1:1 v/v) mixture at a concentration of 1 mg/ml was dropped into 0.75 ml of water. THF was then removed under reduced pressure resulting in the PEGylated SqD–NPs in water at a concentration of 0.5 mg/ml.
- aSq–NPs, which were prepared as follows: 0.1 ml of aSq solution in THF at a concentration of 10 mg/ml was dropped into 1 ml of water under stirring. THF was then removed under reduced pressure resulting in an aSq–NPs suspension in water at a concentration of 1 mg/ml.

The NP characteristics, especially size and polydispersity index (PDI), of the drug-free cSq–NPs and aSq–NPs were studied by varying the initial SqD concentration in THF and the final NP concentration in water. The detailed information is reported in the supplementary information (Supplementary Table 1).

### Physicochemical Characterization of Drug-Free SqD–NPs

#### Size, PDI, Zeta-Potential

The intensity-based hydrodynamic size (reported as *z*-average), PDI, and zeta-potential of the SqD–NPs were determined at 25°C by dynamic and electrophoretic light scattering (DLS, ELS) using a Zetasizer (Zetasizer Nano ZSP, ZEN5600, Malvern, Software 7.02) equipped with a He–Ne Laser at a 633 nm wavelength, backscattering angle of 173° for DLS. For the measurements, 20 μl of SqD–NPs suspension was diluted into 800 μl of water.

#### Morphology

The morphology of each drug-free SqD–NPs was investigated by cryogenic transmission electron microscopy (cryo-TEM). In brief, after plotting 3 μl of SqD–NPs suspension on a holey carbon grid (S147-4, Plano Wetzlar, Germany) for 2 s, the sample was frozen by plunging into −165°C liquid ethane, then transferred to the sample holder under liquid nitrogen conditions. All samples were examined using a JEOL (Akishima, Tokio, Japan) JEM-2100 LaB6 TEM equipped with a Gatan model 914 cryo-TEM sample holder (Pleasonton, CA, USA) and a Gatan Orius SC1000 CCD camera to gain bright-field images. Sample analysis was done at −170°C, under low-dose conditions, meaning conservative settings of ~10 μa/cm^2^ radiation level to avoid sample destruction.

#### Colloidal Stability

The colloidal stability of the drug-free SqD–NPs was studied in physiological relevant pH milieus, including pH 2, pH 5 (acetate buffer solution), and pH 7.4 (HBSS buffer solution). The tested samples were prepared by adding 25 μl of SqD–NPs suspension into 975 μl of buffer solution. NP characteristics, including size, PDI, and zeta-potential, were determined by DLS and ELS using a Zetasizer after 1, 3, and 24 h at 25°C.

### Interaction of SqD–NPs With Biological Systems

#### Biocompatibility

As surrogate for biocompatibility, the determination of cytotoxicity by MTT assay on A549 cells was chosen. Briefly, prior to the assays, 10^4^ cells were seeded in each well of the 96-well plates and grown until reaching 80% cell confluence. The SqD–NPs were suspended in HBSS at concentrations ranging from 0.0652 to 1 mg/ml and incubated with cells for 4 h at 37°C and 5% CO_2_. After the incubation time, cells were washed twice with PBS, and MTT reagent (0.5 mg/ml in HBSS) was added. The cells were then incubated for an additional 4 h, at 37°C, and 5% CO_2_ allowing the formation of formazan crystals intracellularly (Mosmann, 1983). Following the removal of the supernatant, cells and formed formazan crystals were dissolved in DMSO for 30 min, and the absorbance was measured at 550 nm using a Tecan microplate reader Infinite M200Pro (Tecan, Crailsheim, Germany). Cells incubated with 1% Triton TM X-100 in HBSS served as positive controls (0% cell viability), while cells incubated with plain HBSS served as negative controls (100% cell viability), respectively. The percentage of cell viability was calculated relating to the negative controls (Ho et al., 2020).

#### Protein–SqD–NPs Interactions

The interaction between fluorescent SqD–NPs and mucin glycoproteins was studied *via* NTA by NanoSight (LM-10, Laser 532 nm) (Malvern, UK). NTA is able to determine the hydrodynamic diameter of NPs. Under random Brownian motions, NPs scatter light from a laser beam. The scattered light can be visualized by the NTA and captured by a video camera, to enable the software to track the movement of the NPs individually. Using the Stoke–Einstein equation, the hydrodynamic diameter of the particles can be determined.

To study the protein–SqD–NP interaction, the Brownian motion of fluorescent NPs in water was compared to their movement in water containing non-fluorescent 0.1% mucin solution (mucin from porcine stomach, type II, Sigma). For the measurements, used dilutions of the NPs, either in 0.1% mucin solution or water, were 1:100 (aSq–NPs), 1:200 (cSq–NPs), and 1:50 (PEG750Sq–NPs). To detect only the movement of the SqD–NPs, the particles were fluorescently labeled, and the fluorescence mode of the NTA was used. Therefore, SqD–NPs were loaded with 0.5% Nile red by coprecipitation; a detailed description of the method is in the Preparation of Drug-Loaded SqD–NPs section. As a vehicle control sample, the 0.1% aqueous mucin solution was studied with and without the fluorescence filter, to show the ability of the filter to exclude optical interferences of the proteins (Supplementary Figure 9, green line: with filter, gray line: without filter). Videos with a duration of 20–30 s were taken and analyzed by NanoSight Software NTA 3.3. The results of the particle concentration as a function of particle size are reported as mean ± standard error (SE).

### Carrier Properties of SqD–NPs

#### Preparation of Drug-Loaded SqD–NPs

The LC of the synthesized SqDs was investigated using compounds representing different physicochemical properties, namely:

- hydrophobic compounds: cholesteryl BODIPY, Nile red, and dexamethasone,
- hydrophilic and charged compounds: isoniazid, colistin, tigecycline, and FITC-albumin.

With the aim to maximize the LC while maintaining the NP stability, appropriate preparation methods for generating drug-loaded NPs were chosen depending on the drug properties. In brief, we explored three NP preparation methods as follows:

- *Co-precipitation method:* In this study, we found that this method worked best for loading hydrophobic compounds (cholesteryl BODIPY, Nile red, and dexamethasone). Briefly, hydrophobic compound and SqD were dissolved in THF. Upon NP self-assembling in water by nanoprecipitation, the hydrophobic compound localized in the core of the SqD–NPs by hydrophobic interaction.
- *Solvent evaporation method:* In this study, we found that this method worked best for loading hydrophilic compounds having multiple functional groups, especially for small Mw hydrophilic compounds (e.g., isoniazid) and amphiphilic compounds (e.g., colistin). Briefly, the SqD and drug were dissolved completely in the mixture of THF:water (1:1 v/v) at desired concentrations (0.25–0.125 mg NPs/ml of NP suspension). The solution was then stirred for 24 h at room temperature allowing charge interaction of SqD and drug molecules, while the hydrophobic interaction between SqD and drug could be induced upon removal of THF using a rotary evaporator (Rotavapor, Büchi, Essen, Germany) at 40 mbar, 40°C and 65 rpm.
- *Dropping method:* In this study, we found that this method worked best for loading both small (e.g., tigecycline) and high Mw hydrophilic compounds (FITC-albumin) having multiple functional groups. Briefly, the charged drug-free SqD–NPs were prepared in water as described in the Preparation of Drug-Free SqD–NPs section and diluted to a concentration of 0.25 mg/ml. SqD–NPs suspension (1 ml) was then dropped into 1 ml of the prepared drug solution in water under stirring at 200 rpm. The resulting drug-loaded SqD–NPs were further stirred for 1 day at room temperature.

The “solvent evaporation method” and “dropping method,” which allowed the loading of drugs in both compartments, core and shell, of the NPs could be used in multiple drug-loading purposes. As an example, Nile red and FITC-albumin co-loaded cSq–NPs are presented in Supplementary Material Part 4. Briefly, the Nile red-loaded cSq–NPs were prepared by “coprecipitation method” and then dropped into an aqueous solution of FITC-albumin (“dropping method”) allowing the dual-loading of both compounds.

**Figure 2:**
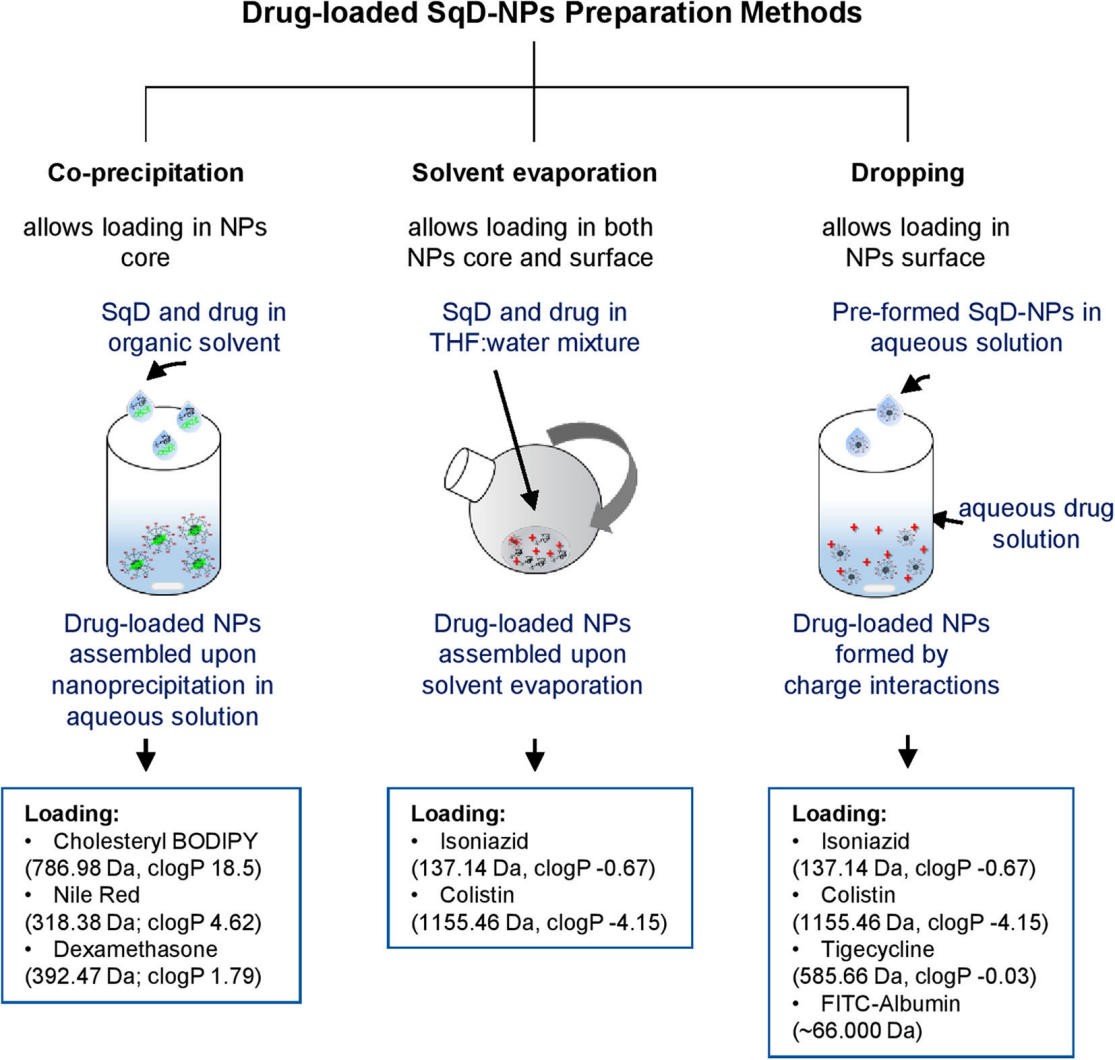
Schematic illustration of drug-loaded SqD–NPs preparation methods and model compounds with corresponding molecular weight and clog *P*-values (estimated by ChemDraw Professional 16.0).

Figure 2 summarizes an overview of the preparation methods for drug-loaded SqD–NPs, listing all the model cargos with corresponding Mw and clog *P*-values (estimated by ChemDraw Professional 16.0).

##### Quantification of loading capacity and encapsulation efficiency

The loading capacity (LC) and encapsulation efficiency (EE) of the drugs loaded to SqD–NPs were determined indirectly using the amount of non-loaded drug in the supernatant of the SqD–NPs suspension. The hydrophobic compound (dexamethasone, cholesteryl BODIPY, or Nile red) was extracted from the supernatant using ethyl acetate (Ho et al., 2018). The hydrophilic compound was analyzed in the supernatant after centrifugation of the SqD–NPs suspension. Isoniazid was collected by loading the NPs in a Centrisart filter (MWCO 10.000 Da) (Sartorius, Göttingen, Germany) and centrifuging at 2,000 *g* for 30 min. Colistin, tigecycline, or FITC-albumin was collected by centrifuging the drug-loaded NPs at 24.400 *g* for 30 min. The amount of cholesteryl BODIPY, Nile red, tigecycline, or FITC-albumin in the supernatant, respectively, was analyzed by a plate reader (details described in Supplementary Material Part 6), while dexamethasone, isoniazid, or colistin were quantified by high-performance liquid chromatography (HPLC) (details described in Supplementary Material Part 6).

(1) $$\begin{array}{r} EE\;\left[\%\right]=\frac{drug\;amount\;loaded\;in\;NPs\;\left[mg\right]}{initial\;drug\;amount\;\left[mg\right]}\;*\;100 \end{array}$$

(2) $$\begin{array}{r} LC\;\left[\%\right]=\frac{drug\;amount\;loaded\;in\;NPs\;\left[mg\right]}{total\;amount\;of\;NPs\;\left[mg\right]}\;*\;100 \end{array}$$

The drug amount loaded in NPs is calculated by subtraction of the non-loaded drug amount from the initial one. The total amount of NPs equals the sum of the amount of SqD and the amount of drug, which is loaded to the NPs.

#### Release Studies

The release studies of selected drug-loaded SqD–NPs were performed using the same procedure in PBS (pH 7.4) at 37°C and constant shaking at 250 rpm. Briefly, the optimal LC sample of the drug-loaded SqD–NPs was concentrated and then diluted in PBS to have a final concentration of the corresponding drug at 10% w/w. The cumulative drug release in percent was evaluated over a 24 h period. Samples were collected after 1, 2, 4, 6, 8, 16, and 24 h, while the release acceptor volume was always kept constant. The drug amount in the acceptor fluid was analyzed. The hydrophobic drugs were extracted using ethyl acetate before further analysis. The drug quantification was done by plate reader or HPLC (Supplementary Material Part 6).

### Statistics

If not stated otherwise, all procedures were conducted at least in three independent experiments and measured in technical triplicate. Results are presented as mean ± standard deviation (SD). Calculations were done using either Excel, Microsoft 2016 and 2019, or GraphPad Prism 8.0. Physicochemical information of molecules was predicted by ChemDraw Professional 16.0.

## Results and Discussion

### Synthesis and Characterization of SqDs

The cSq and PEGylated SqDs were straightforwardly obtained by simple reductive amination reaction from 1,1′,2-trisnorsqualenic aldehyde. The successful synthesis and purification of all SqDs were confirmed by ^1^H-NMR and ^13^C-NMR (Supplementary Figures 1–8). These results were confirmed by MS for aSq [(ESI-) *m/z(%)* 465.3 (100) (M-H)^−^] and cSq [(ESI-) *m/z(%)* 474.4 (100) (M-H)^+^], respectively. The synthetic schemes are facile and allowed isolating the final products in high yields (>90%), which, moreover, could be scaled-up easily. Notably, the synthesis of cSq with a diethanolamino polar head was further optimized with one step less compared to that of the previously reported squalene-amine derivative (van Tamelen and Curphey, 1962; Ralay-Ranaivo et al., 2014).

### Preparation and Physicochemical Characterization of Drug-Free SqD–NPs

#### Size, PDI, Zeta-Potential, and Morphology

**Figure 3:**
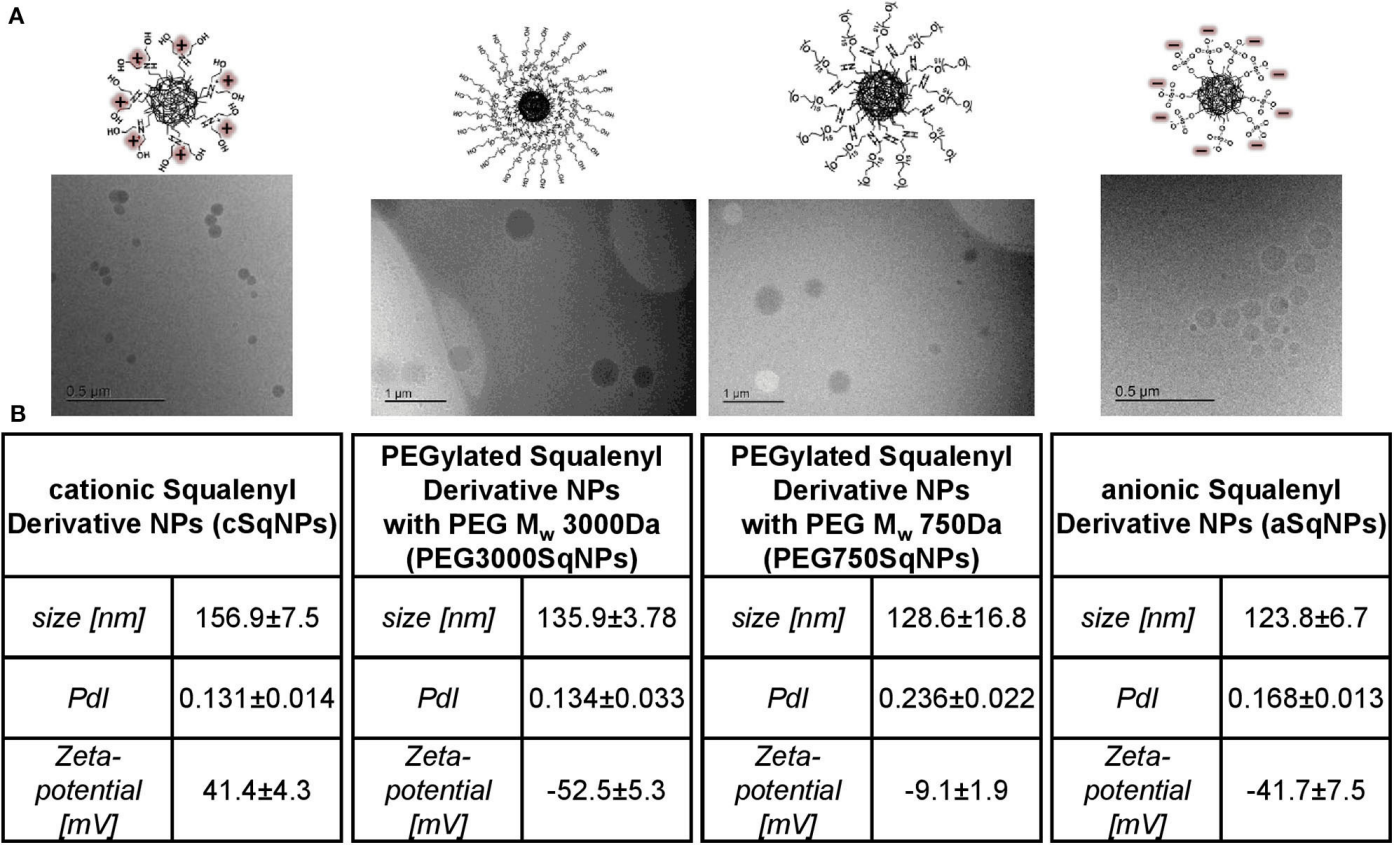
Characterization of SqD–NPs, from left to right: cSq–NPs, PEG3000Sq–NPs, PEG750Sq–NPs, aSq–NPs. **(A)** Cryo-TEM images of SqD–NPs. **(B)** Hydrodynamic size, polydispersity index (PDI), zeta-potential determination by dynamic light scattering (DLS), and electrophoretic light scattering (ELS). Results presented as mean ± SD.

Overall, the introduction of anionic, cationic, or PEG moiety into squalene enhanced the amphiphilic properties of the synthesized SqDs and enabled the facile self-assembly into uniform and stable NPs in aqueous solution. Regardless of the SqD, the drug-free NPs had mean sizes ranging from 100 to 200 nm and a narrow size distribution (PDI < 0.25) (Figure 3B, Supplementary Table 1). The zeta-potential of aSq–NPs and cSq–NPs were around −40 and +40 mV, respectively, indicating the presence of their corresponding charged groups on the NP surface (Figure 3B, Supplementary Table 1). Such strong charges facilitate the further drug loading on the surface *via* electrostatic interaction and enable NP stability (Bhattacharjee, 2016).

We prepared two PEGylated SqDs having PEG with different chain length and terminal groups. The PEG750Sq terminates with a methyl group, while the PEG3000Sq terminates with a hydroxyl group. The self-assembling of these PEGylated SqDs allowed the formation of NPs with a dense surface layer of PEG. Regardless of the recorded zeta-potential, this PEG layer is expected to stabilize the NPs and minimize surface interactions between NPs and other molecules (Suk et al., 2016).

Different initial concentrations of aSq in THF could slightly tune the size of aSq–NPs that were prepared by nanoprecipitation in water at a final concentration of 1 mg/ml (Supplementary Table 1). By increasing the aSq concentration in THF from 2.5 to 25 mg/ml, the aSq-NPs had the size raised from 90 to 160 nm. This phenomenon could be explained by the assembly of more squalenyl moieties in the core compartment at higher concentrations (Ho et al., 2018). In contrast, varying the final aSq-NPs concentration in water and using the same initial aSq concentration in THF did not influence the NP size (Supplementary Table 1). A similar observation was reported previously for squalenoylated chitosan NPs, which showed an increase rather in NP count than in NP size (Lepeltier et al., 2015). The drug-free cSq–NPs, in turn, could be only formed in the size range of 200 nm regardless of the variation of either initial cSq concentration in organic solvent or final NP concentration in water. The cryo-TEM images of the drug-free SqD–NPs are presented in Figure 3A illustrating the amorphous and spherical-shaped NPs. No differences in morphology between the SqD–NPs were observed.

#### Colloidal Stability

**Figure 4:**
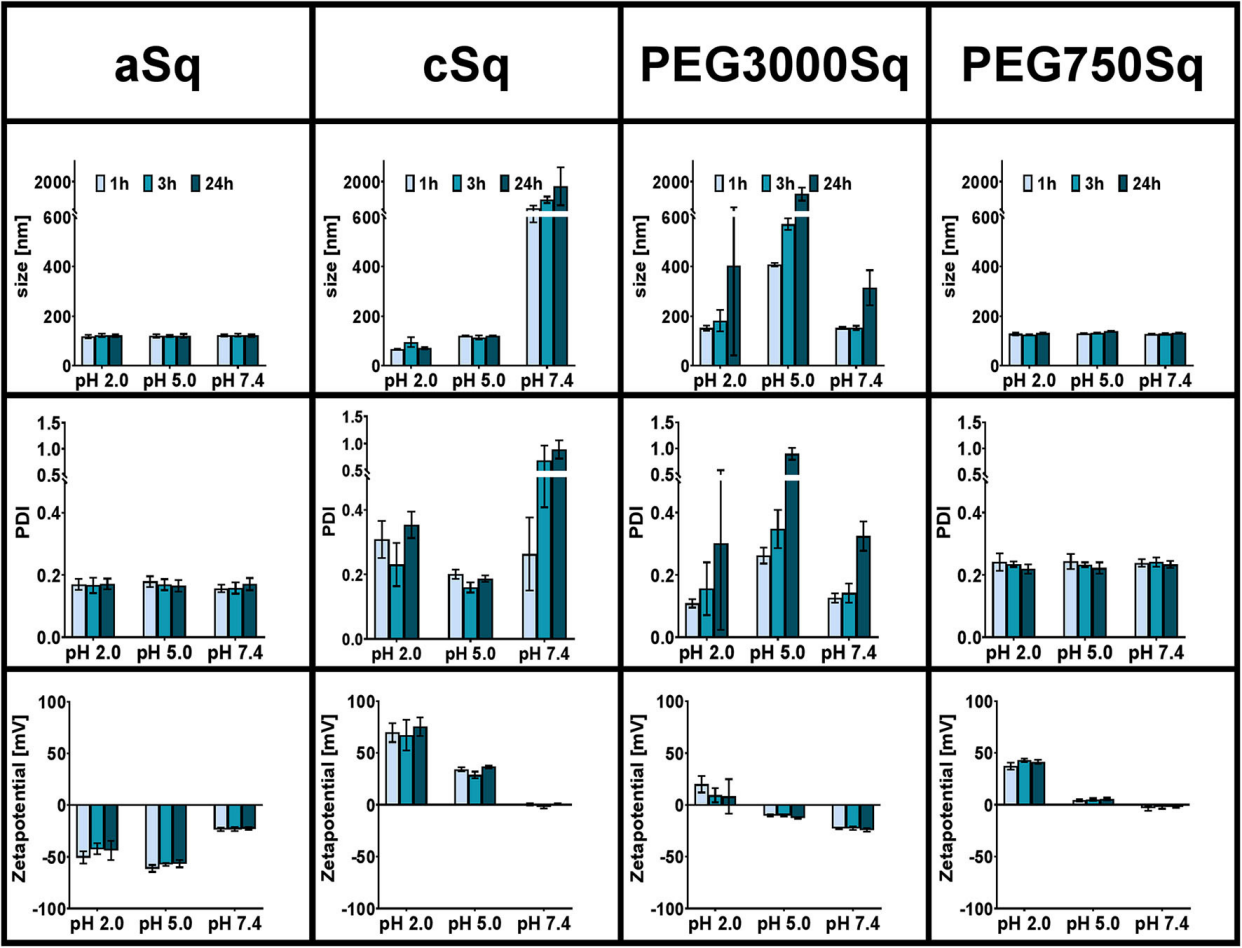
Colloidal stability of SqD–NPs in different physiological relevant pH milieus (pH 2, 5, 7.4) at room temperature studied by DLS and ELS. Hydrodynamic size, PDI, and zeta-potential are reported as mean ± SD. Measurements were taken at three different time points: 1 h (light blue bars), 3 h (blue bars), 24 h (dark blue bars).

The colloidal stability of drug-free SqD–NPs was investigated in different pH values representing gastric acid (pH 1–3), skin (pH ~5), and common physiological pH environments at 7.4 (Schmid-Wendtner and Korting, 2006; Koziolek et al., 2015). The colloidal stability in the corresponding pH was evaluated based on the changes of NP size, PDI, and zeta-potential at 1, 3, and 24 h post-incubation. Overall, the aSq–NPs and PEG750Sq–NPs were stable after 24 h of incubation in all studied pH milieus, while the stability of cSq–NPs could only be maintained at pH 5 or lower. In contrast, the incubation of PEG3000Sq–NPs in all tested buffers resulted in colloidal aggregation over time (Figure 4).

The hydrogen sulfate groups on aSq-NPs surface were strongly acidic and deprotonated at all tested pH values resulting in a low zeta-potential (around −50 and −25 mV in pH 2/5 and 7.4, respectively), which helped stabilizing the NP suspension (Bhattacharjee, 2016). At 24 h post-incubation in all conditions, the aSq–NPs hydrodynamic diameter was always around 120 nm, while the PDI was lower than 0.2.

The cSq–NPs, in turn, exposing the diethanolamine moiety on its surface, in which pK_a_-value is estimated at 8.44 (ChemDraw Professional 16.0), showed positive zeta-potential of 71 ± 11 and 33 ± 4 mV at pH 2 and 5, respectively, reflecting the protonation of the amine groups. The size and PDI of stable cSq–NPs after 24 h of incubation at pH 5 were 120.5 ± 1.5 nm and 0.19 ± 0.01 nm, respectively. Interestingly, a slightly smaller particle size (76.9 ± 17.1 nm) and a larger PDI (0.30 ± 0.07) were recorded when dispersing cSq–NPs at pH 2. The cSq–NPs could be fully charged at pH 2, which enhanced the amphiphilicity of cSq molecules. Consequently, this induced intermolecular forces and hydrophobic interactions between the squalenyl moieties leading to a denser particle packing (Ho et al., 2018). The same phenomenon was also observed before in farnesylated glycol chitosan NPs (Ho et al., 2018). At neutral pH 7.4, amine functional groups might become partly uncharged causing a decrease in zeta-potential from ~70 mV at pH 2 to around zero, and the aggregation of cSq–NPs was observed. The colloidal instability of cSq–NPs at pH 7.4 makes these DDS rather unfavorable for systemic drug delivery. However, cSq–NPs demonstrate a good colloidal stability at pH 5, which represents the pH milieu of the skin barrier (Schmid-Wendtner and Korting, 2006). Topical application of NPs to the skin surface are known to enhance the follicular drug delivery. cSq-NPs could be especially further explored to be used for follicular vaccination (Mittal et al., 2015). In addition to the possible complexation of DNA, squalene was already used in adjuvants, boosting an immune response against vaccine antigens. Even the *in vitro* and *in vivo* transfection efficiency of a DNA complex emulsion was improved in the presence of squalene compared to the control complexes (Kwon et al., 2008; Reddy and Couvreur, 2009).

PEGylation offers plenty of advantages to nano-sized drug carriers especially to improve the *in vivo* stability and therapeutic efficacy (Suk et al., 2016; Thomas and Weber, 2019). Previously, PEGylated SqD with a Mw of 1,955 Da was incorporated into squalenoylated gemcitabine prodrug NPs in order to enhance their stability at pH 7.4 and gemcitabine performance (Bekkara-Aounallah et al., 2008). In this study, we aim to investigate the potential of PEGylated SqDs as a platform for drug delivery. Hence, the stability of NPs assembled from PEG3000Sq or PEG750Sq was studied individually. As shown in Figure 4, PEG3000Sq–NPs were unstable in all tested buffer media making it unfavorable for use as DDS. Although the small molecule squalene (*Mw* = 410 Da) holds excellent assembling properties, the monoconjugation with PEG having a Mw of 3,000 Da might result in a better water solubility. Thus, the hydrophobic interaction of squalenyl moieties would not be strong enough to stabilize the PEG3000Sq–NPs. On the complete opposite, SqPEG750–NPs demonstrated excellent colloidal stability in all pH milieus at all time points. The hydrodynamic diameter and PDI were always around 129.7 ± 4.5 nm and lower than 0.23 ± 0.02, respectively. The zeta-potential of SqPEG750–NPs was nearly neutral at pH 5 and 7.4; however, it increased to 40 ± 3 mV at pH 2 due to the protonation of the amino conjugation.

The particles holding appropriate colloidal stability in physiological relevant pH milieus were used in further investigations, including aSq–NPs, cSq–NPs, and PEG750Sq–NPs.

### Interaction of SqD–NPs With Biological Systems

#### Biocompatibility

**Figure 5:**
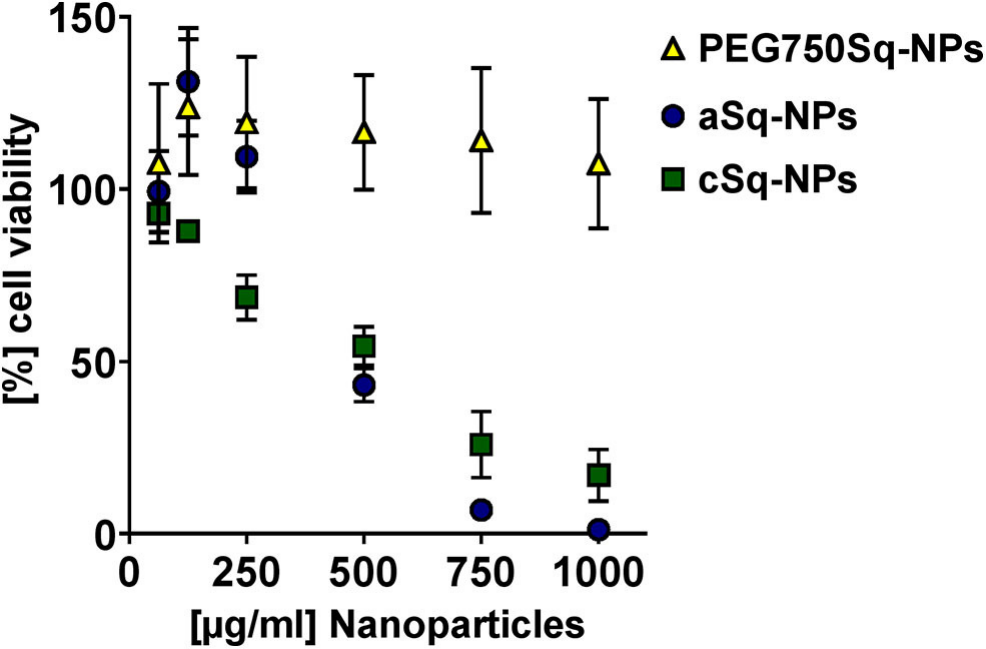
Biocompatibility study *via* MTT assay at different concentrations of PEG750Sq–NPs (yellow triangle), aSq–NPs (dark blue circles), and cSq–NPs (green squares) diluted in HBSS on A549 cells over 4 h at 37°C and 5% CO_2_. Results are presented as (%) cell viability relating to negative control in plain HBSS at different NP concentrations and reported as mean ± SD.

The biocompatibility of the SqD–NPs was tested on A549 cells *via* MTT assay (Figure 5). In good agreement with Ho et al. (2020) aSq–NPs demonstrated 100% viability for up to 250 μg/ml (blue circles in Figure 5). The cSq–NPs (green squares in Figure 5) showed slightly lower tolerability than aSq-NPs, which might have an explanation in the instability of cSq–NPs in HBSS buffer causing large aggregates on cells. Moreover, the nature of a cationic lipid can enhance the toxicity due to its potential interaction with anionic biological molecules like proteins and nucleic acids (Knudsen et al., 2015). Thus, only 70% cell viability was found when incubating cSq–NPs at a concentration of 250 μg/ml. Similar findings were previously described: the incubation of 250 μg/ml of positively charged squalenoylated chitosan NPs and farnesylated glycol chitosan NPs resulting in ~80 and >70% A549 cell viability, respectively (Lepeltier et al., 2015; Ho et al., 2018). The PEG750Sq–NPs (yellow squares in Figure 5) were highly biocompatible demonstrating 100% cell viability at the highest tested concentration of 1 mg/ml.

#### Protein–SqD–NPs Interactions

**Figure 6:**
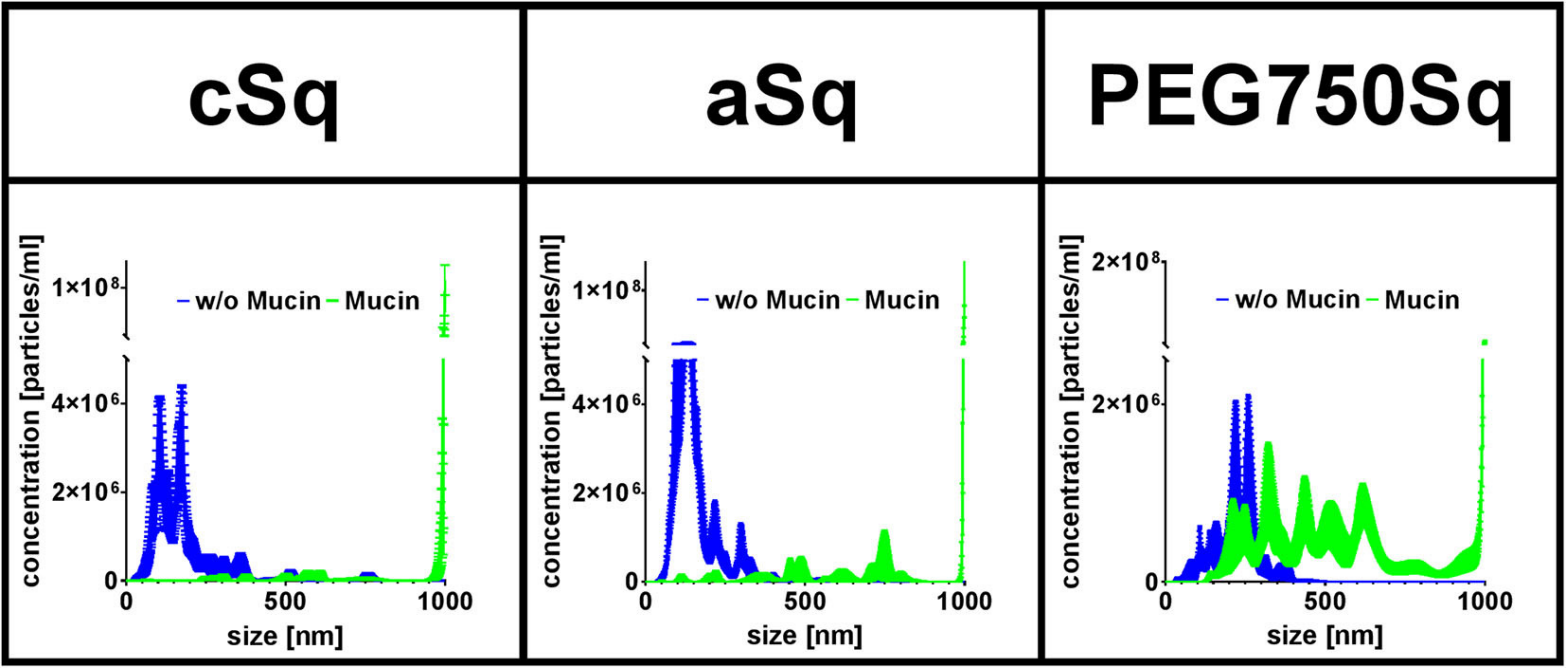
Fluorescent SqD–NPs interaction study with mucin glycoproteins. Interactions detected by size shift determined by nanoparticle tracking analysis (NTA) measurements. Blue lines: measurement of fluorescent SqD–NPs in water. Green lines: measurement of fluorescent SqD–NPs in non-fluorescent 0.1% mucin glycoprotein aqueous solution. Results are presented as mean ± SE.

Exemplary for a protein interaction study, the physicochemical interactions between fluorescent SqD–NPs and mucin glycoproteins were studied using NTA. The hydrodynamic size of the SqD–NP suspension was compared either in (i) water (blue line in Figure 6) or in (ii) 0.1% mucin glycoprotein aqueous solution (green line in Figure 6). It was hypothesized that non-interacting, fluorescent SqD–NPs would display the same particle size distribution irrespective of dispersion in water or in the low-viscosity mucin glycoprotein-containing solution. Conversely, the fluorescence signal of SqD–NPs interacting with non-fluorescent mucin glycoproteins would display a different Brownian motion, indicative of a movement hindrance, and resulting in a particle size distribution shifted toward larger particle diameters. By comparing the results of the measurements of SqD–NPs in both aqueous solution (Figure 6) on this basis, the interaction between the mucin glycoproteins and NPs can be ranked from high to low as follows: cSq–NPs > aSq–NPs >> PEG750Sq–NPs.

In detail, cSq–NPs were found to present a highly positive surface net charge enabling interaction with the negatively charged mucin glycoproteins. These strong interactions are demonstrated by a size shift to a larger size from ~180 to ~960 nm (mean of number–weighted distribution) (blue and green lines in Figure 6, respectively). Further, the negatively charged aSq–NPs showed interactions with the mucin glycoproteins, shifting the mean particle size from ~160 to ~930 nm (blue and green lines in Figure 6, respectively), which is less pronounced than for cSq–NPs. We assumed that those interactions are mainly caused by the hydrophobic squalenyl moiety (Murgia et al., 2018). As expected, PEG750Sq–NPs demonstrated only minor interactions with mucin glycoproteins (mean size shift from ~230 to ~575 nm, blue and green lines in Figure 6, respectively). Still PEG750Sq–NPs resulted in partial mucin adsorption, which can be explained by hydrophobic interactions between mucin glycoproteins and the NP core.

Our results, showing that interactions between mucin glycoproteins and charged NPs, cSq–NPs, and aSq–NPs are more pronounced than interactions with neutral PEG750Sq–NPs, are in good concordance with previous findings (Crater and Carrier, 2010). PEGylated NPs, with a sufficiently high PEG density on the NP surface, are widely known to be mucus penetrating because they are inert to hydrophobic interactions and hydrogen bonding (Wang et al., 2008; Xu et al., 2015; Maisel et al., 2016).

### Carrier Properties of SqD–NPs

#### Optimal Loading Capacities of SqD–NPs

We investigated the LC of the SqD–NPs using a variety of compounds owning multiple functional groups as well as representing different physicochemical characteristics and Mw. The suitable SqD and drug-loading method to obtain optimal LC for each model compound are reported in Table 1, while detailed guidance for choosing appropriate SqD and preparation method is shown in Figure 2. Generally, the SqD–NPs are composed of two compartments—a hydrophilic shell and a hydrophobic core. The hydrophobic drugs were loaded in the core by hydrophobic interactions *via* the coprecipitation method or the solvent evaporation method. For the hydrophobic compounds, e.g., Nile red, cholesteryl BODIPY, or dexamethasone, the coprecipitation method offers more facile loading procedure and better LC. The hydrophilic drugs, in turn, could be loaded onto the NP shell by charge interactions and hydrogen bonding *via* the dropping method or the solvent evaporation method. Interestingly, by allowing the drug to distribute in both the lipophilic and hydrophilic domains of the squalenyl nanoassemblies, the solvent evaporation method is a good loading technique for amphiphilic compounds, e.g., colistin.

**Table 1:** Summary of preparation methods used to load model compounds to squalenyl derivative–nanoparticles (SqD-NPs).

**Squalenyl-derivative**	**Drug**	**Preparation method**	**Size (nm)**	**PDI**	**Zeta-potential (mV)**	**EE(%)**	**LC (%)**
aSq	Cholesteryl BODIPY	Coprecipitation	254.3 ± 2.4	0.094 ± 0.011	−18.6 ± 0.1	87.6 ± 8.2	10.35 ± 0.66
	Dexamethasone	Coprecipitation	140.2 ± 25.7	0.203 ± 0.061	−40.0 ± 7.1	90.6 ± 1.2	32.75 ± 0.33
	Isoniazid	Solvent evaporation	198.9 ± 8.6	0.249 ± 0.029	−29.5 ± 1.0	42.6 ± 1.7	27.43 ± 0.79
		Dropping	112.9 ± 2.0	0.125 ± 0.017	−35.6 ± 2.3	07.4 ± 1.2	06.17 ± 0.97
	Colistin	Solvent evaporation	325.0 ± 7.1	0.152 ± 0.041	22.9 ± 1.1	90.1 ± 2.3	45.31 ± 0.73
		Dropping	195.6 ± 2.6	0.184 ± 0.002	25.1 ± 1.0	83.6 ± 4.1	35.83 ± 0.51
	Tigecycline	Dropping	198.6 ± 4.8	0.014 ± 0.010	28.6 ± 1.3	85.3 ± 6.7	44.26 ± 2.03
cSq	Nile Red	Coprecipitation	208.0 ± 3.2	0.185 ± 0.069	38.6 ± 3.7	60.2 ± 6.7	09.03 ± 0.91
	Dexamethasone	Coprecipitation	146.9 ± 8.9	0.183 ± 0.027	44.1 ± 5.7	87.9 ± 6.6	32.09 ± 1.64
	Fluorescent Albumin	Dropping	205.6 ± 2.8	0.174 ± 0.010	17.4 ± 1.7	92.1 ± 3.8	03.52 ± 0.33
PEG750Sq	Dexamethasone	Coprecipitation	162.95 ± 59.45	0.255 ± 0.075	00.1 ± 9.2	92.4 ± 2.5	23.49 ± 0.48

*The characteristics of optimal drug-loaded SqD–NPs, including hydrodynamic size, polydispersity index (PDI), zeta-potential, encapsulation efficiency (EE), and loading capacity (LC) are reported. Results are presented as mean ± SD*.

In this study, the model compounds dexamethasone, cholesteryl BODIPY, and Nile red—representing different Mw and hydrophobicity—could be loaded in the core of any SqD–NPs. The drug-loaded NPs had a size range of 130–250 nm and a narrow size distribution (PDI < 0.3). Notably, the optimal LC of dexamethasone was ~33, ~32, and ~24% in aSq–NPs, cSq–NPs, and PEG750Sq–NPs, respectively, while the EE values in all cases were ~90% (Table 1). The optimal LC and EE of cholesteryl BODIPY in aSq–NPs were 10.35 ± 0.66% and 87.6 ± 8.2%, respectively. The optimal LC and EE of Nile red in cSq–NPs were 9.03 ± 0.91% and 60.2 ± 6.7%, respectively. The lower EE recorded in loading Nile red could be explained by its low Mw and less interaction with the squalenyl core. Squalene is known as a precursor in the endogenous cholesterol biosynthesis, which implies its strong interaction with dexamethasone—a derivative of cholesterol—thereof enhancing the LC and EE (Reddy and Couvreur, 2009; Ghimire et al., 2016). The same holds true for cholesteryl BODIPY, which has a high clog *P*-value and contains a cholesteryl moiety.

Isoniazid, colistin, tigecycline, and FITC-albumin, representing different Mw and hydrophilicity, were loaded in either aSq–NPs or cSq–NPs. Accordingly, the loading of these compounds was investigated using solvent evaporation method and/or dropping method (Figure 2), while the optimal LC and EE for each compound and the corresponding loading method are reported in Table 1.

Isoniazid—a positively charged and small molecule (Mw 137.14 Da)—was loaded in aSq–NPs. The solvent evaporation method resulted in a significantly higher LC of isoniazid at 27.43 ± 0.79% compared to that of the dropping method at 6.17 ± 0.97%. Using the solvent evaporation method, the preparation of isoniazid and aSq in the solvent mixture (THF:water 1:1 v/v) allowed the maximum charged interaction between the two reagents, thus, increased the EE and LC. In contrast, the preformed aSq–NPs and dropping method limited the interaction with isoniazid molecules. The optimal isoniazid-loaded aSq–NPs had the size of 198.9 ± 8.6 nm and PDI below 0.25.

The molecules with multiple functional groups—tigecycline (Mw 585.66 Da), colistin (Mw 1,155.46 Da), and FITC-albumin (Mw ~60,000 Da)—could be loaded in NPs using the dropping method. The optimal LC and EE of stable tigecycline-loaded aSq–NPs (198.6 ± 4.8 nm, PDI 0.014 ± 0.010) were 44.26 ± 2.03 and 85.3 ± 6.7%, respectively. The LC and EE of colistin in aSq–NPs were significantly high at 35.83 ± 0.51 and 83.6 ± 4.1%, respectively, which also produced a stable DDS having the size of 195.6 ± 2.6 nm and PDI lower than 0.2. The reasonable LC and EE of FITC-albumin—a representative of negatively charged protein molecules—in cSq–NPs were 3.52 ± 0.33 and 92.1 ± 3.8%, respectively. The stable protein-loaded cSq–NPs had the size of 205.6 ± 2.8 nm and PDI below 0.2. As shown in Table 1, the zeta-potential values of these drug-loaded NPs changed significantly compared to the corresponding drug-free NPs reflecting the loading *via* charged interactions.

The positively charged colistin contains a lipophilic moiety and could interact with aSq molecules *via* both charged and hydrophobic interactions (Yasar et al., 2018; Menina et al., 2019). We, thus, hypothesized that the solvent evaporation method could enhance the LC and EE of colistin in aSq–NPs. In fact, colistin LC and EE obtained by using such an approach were 45.31 ± 0.73 and 90.1 ± 2.3%, respectively, which were higher than the ones obtained by the dropping method. The integration of the lipophilic moiety of colistin into aSq–NPs increased the NP size from 195.6 ± 2.6 to 325.0 ± 7.1 nm, while the positive zeta-potential (22.9 ± 1.1 mV) of the drug-loaded aSq–NPs indicated also the presence of colistin on the NP surface.

The dual loading capacity of the SqD–NPs was illustrated by loading both hydrophobic Nile red and hydrophilic FTIC-albumin in cSq-NPs. The Nile red-loaded cSq–NPs (LC ~0.5%) were prepared by the coprecipitation, and then further loaded with hydrophilic FTIC-albumin (LC~0.5%) by the dropping method. The colocalization of both fluorescent compounds in NPs was confirmed by confocal laser scanning microscopy images (Supplementary Figure 10). Such simultaneous drug loadings could benefit the delivery of actives with synergistic effects (Ho et al., 2020).

In short, the SqD–NPs are capable of loading a broad range of drug molecules differing in physicochemical characteristics, thus, are versatile drug delivery platforms.

#### Release Studies

**Figure 7:**
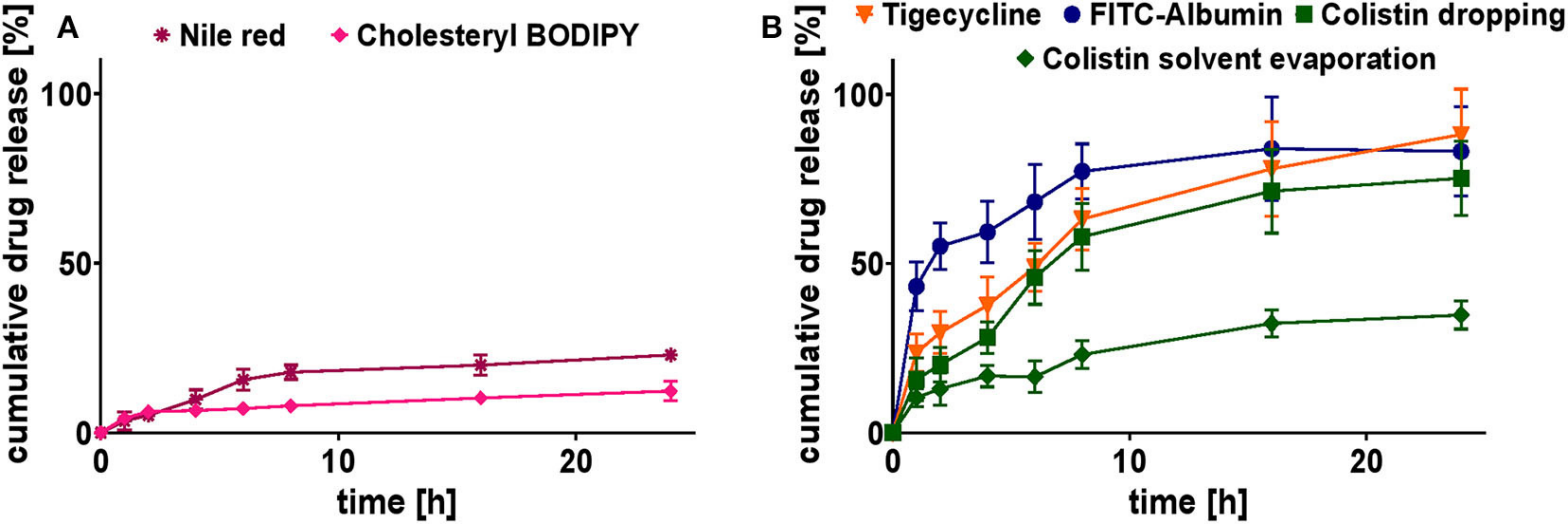
Cumulative release study of selected model drug-loaded SqD–NPs at pH 7.4 and 37°C for 24 h in PBS. **(A)** Cumulative release profiles of hydrophobic compounds: Nile red (purple star), cholesteryl-BODIPY (pink rhomb). **(B)** Cumulative release profiles of hydrophilic compounds: FITC-albumin (blue circles), tigecycline (orange triangle), colistin (dropping: green square; solvent evaporation: green rhomb). Results are presented as mean ± SD.

*In vitro* release profiles were conducted under the same conditions at physiological pH 7.4 (PBS), 37°C, and over 24 h (Figures 7A,B).

The release profiles of hydrophobic compounds were determined for the ones with the highest and lowest Mw, cholesteryl BODIPY, and Nile red, respectively. The release profiles for both compounds are shown in Figure 7A. They clearly demonstrate their affection by the strength of hydrophobic interactions. Compared to cholesteryl BODIPY, Nile red released twice the cumulative percentage after 24 h. Nevertheless, the release of both compounds was sustained for the period over at least 24 h.

The release profiles of hydrophilic compounds were conducted for tigecycline (highest achieved LC), FITC-albumin (protein representative) and colistin (comparison of two preparation methods), respectively. The association of hydrophilic drugs loaded onto the NP surface by the dropping method is not as strong as a covalent conjugation, leading to the assumption of a complete drug release. Cumulative release of tigecycline, colistin, and FITC-albumin reached 88 ± 13, 75 ± 11, and 83 ± 13%, respectively, after 24 h in PBS at 37°C (Figure 7B). The burst release at the early time points were avoided suggesting the strong charged interactions between the SqD and drug molecules. In comparison to that, the tight interaction of colistin to aSq–NPs by using the solvent evaporation method resulted in a sustained release for colistin. Only 35 ± 4% of colistin was released from aSq–NPs after 24 h in PBS at 37°C (Figure 7B).

Furthermore, IC90 values of tigecycline and colistin-loaded aSq–NPs were determined by the minimum inhibitory concentration (MIC) assay performed on *Pseudomonas aeruginosa* strain PA14 wild type and *Staphylococcus aureus* strain Newman. As shown in Supplementary Table 3, the antimicrobial effect of both antibiotic-loaded NPs was similar to that of the drug alone, even though drug release after 24 h from the NPs is not completed.

## Conclusion

In this study, we explored the use of self-assembling amphiphilic SqDs as DDS, which had started with the aSq. We have further described the straightforward synthesis of cSq and PEGylated SqDs. In addition to the ability of self-assembling to supramolecular colloids, the latter demonstrated that SqD–NPs showed excellent stability in physiological relevant media and biocompatibility. The application of different methods for the preparation of drug-loaded SqD–NPs allowed to modulate not only the LC but also the release rate, as desired. In further studies of carrier properties, the SqD–NPs showed significantly high LC of various cargos. Our findings postulate the self-assembled SqD–NPs as versatile drug delivery platforms.

## Data Availability Statement

The raw data supporting the conclusions of this article will be made available by the authors, without undue reservation.

## Author Contributions

D-KH, BL, DD, PC, and C-ML conceptualized and initiated the study. PC and C-ML acquired funding and provided resources. XM, US, BL, DD, PC, and C-ML were supervisors. D-KH did chemical synthesis and characterization. D-KH and RC investigated NP self-assembly and drug-loading capacities. CDR, RC, and D-KH validated the drug quantification methods. D-KH conducted *in vitro* assays and drug release. CDR and MK visualized the NPs. XM conceptualized and validated the mucin–NPs interaction studies. RC and SF performed NP stability and mucin–NPs interaction studies. RC and D-KH analyzed the data, prepared the figures, and wrote the original draft. RC reviewed and edited the final draft. All authors read and revised the manuscript.

## Conflict of Interest

The authors declare that the research was conducted in the absence of any commercial or financial relationships that could be construed as a potential conflict of interest.

## Abbreviations

LC: drug loading capacity
SqD: squalenyl derivative
aSq: anionic squalenyl derivative
cSq: cationic squalenyl derivative
PEG750Sq: PEG750 squalenyl derivative
PEG3000Sq: PEG3000 squalenyl derivative
NP: nanoparticle
NTA: nanoparticle tracking analysis
DLS: dynamic light scattering
ELS: electrophoretic light scattering
cryo-TEM: cryogenic transmission electron microscopy
MS: mass spectrometry
PDI: polydispersity index
HPLC: high performance liquid chromatography
SD: standard deviation
SE: standard error.
