# Supplementary material for: Synthesis and Biopharmaceutical Characterization of Amphiphilic Squalenyl Derivative Based Versatile Drug Delivery Platform
Source: Front Chem. 2020 Oct 19;8:584242. doi: 10.3389/fchem.2020.584242 (PMC7604382; doi:10.3389/fchem.2020.584242)
Supplement: Supplementary file 1 [file Data_Sheet_1.PDF]

Supplementary Materials

1 ^{1}H- & ^{13}C-NMR Spectra

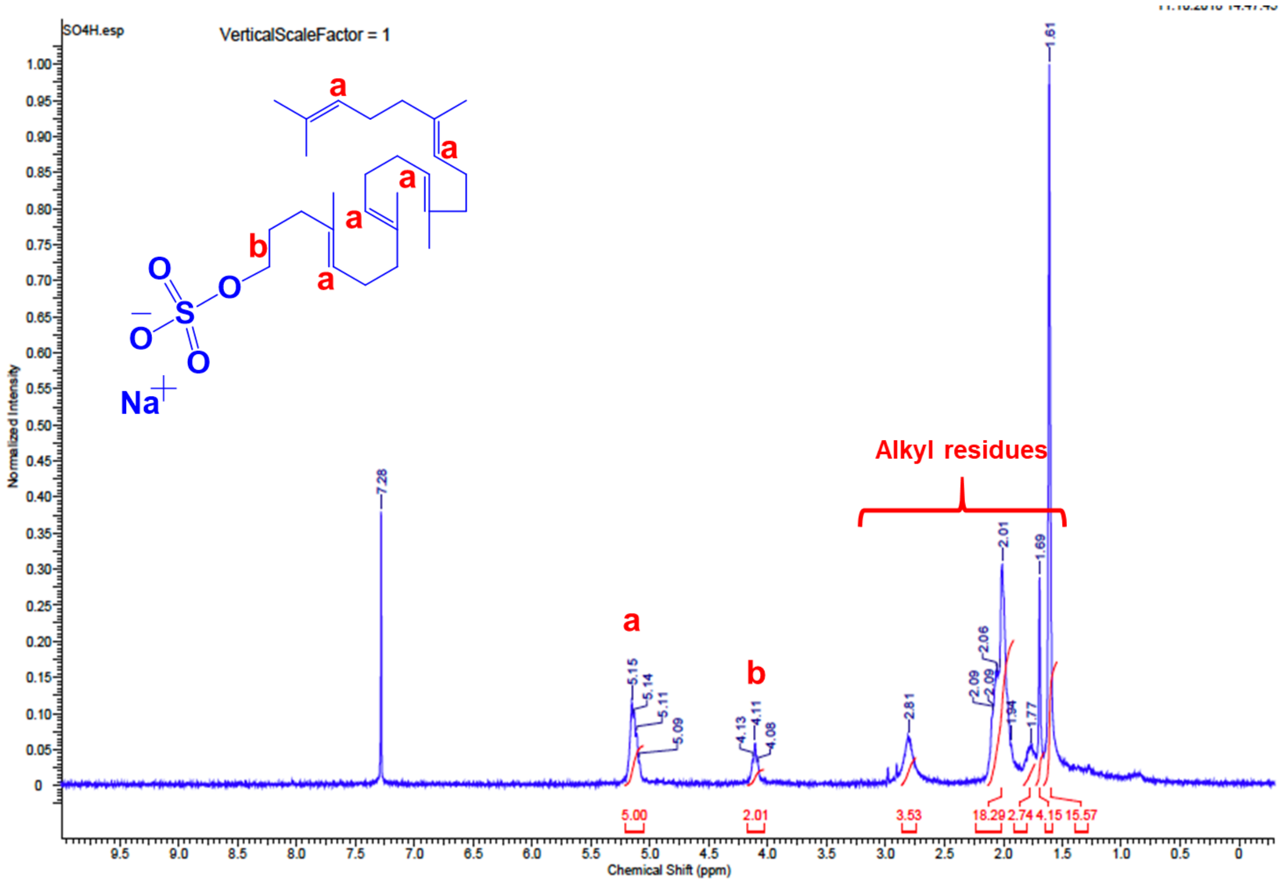

Figure S1 ^{1}H-NMR Spectrum aSq

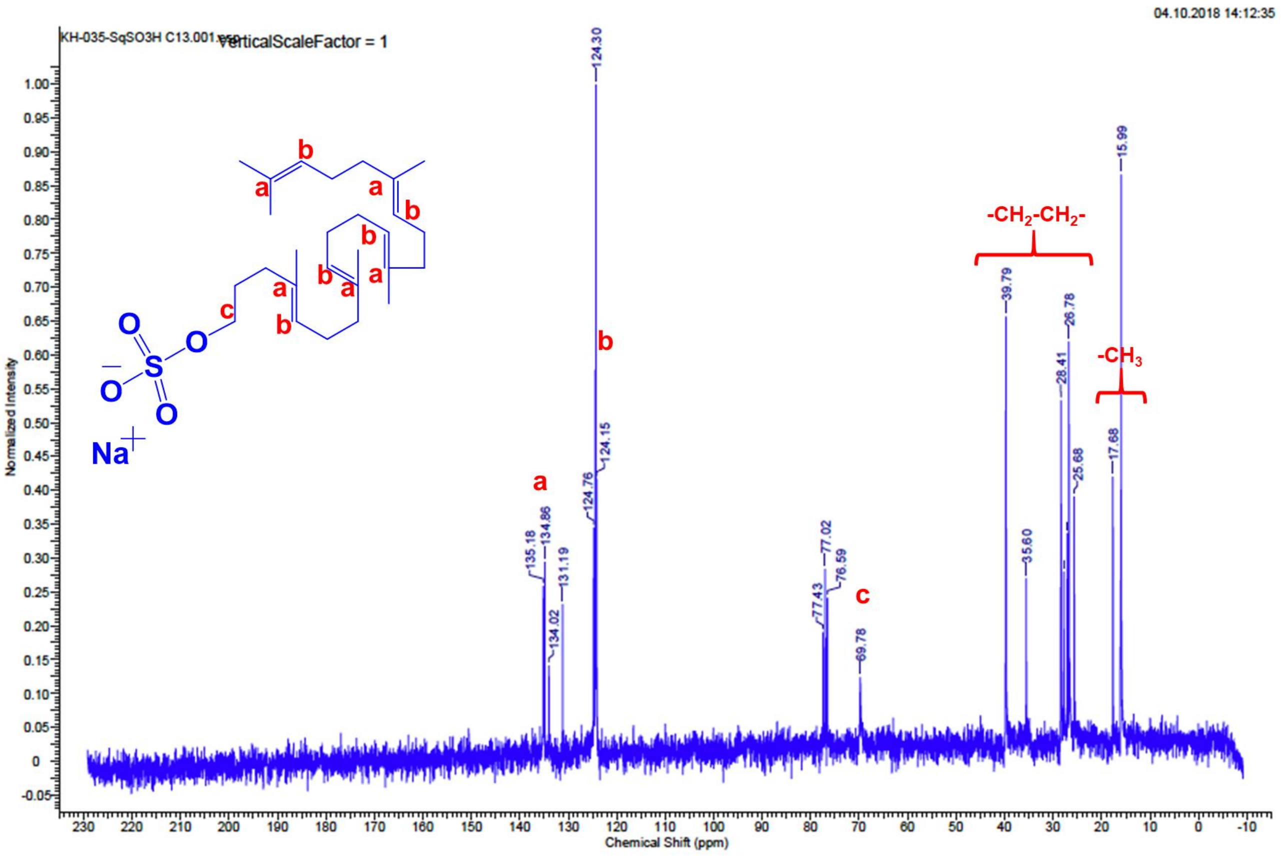

Figure S2 ^{13}C-NMR Spectrum aSq

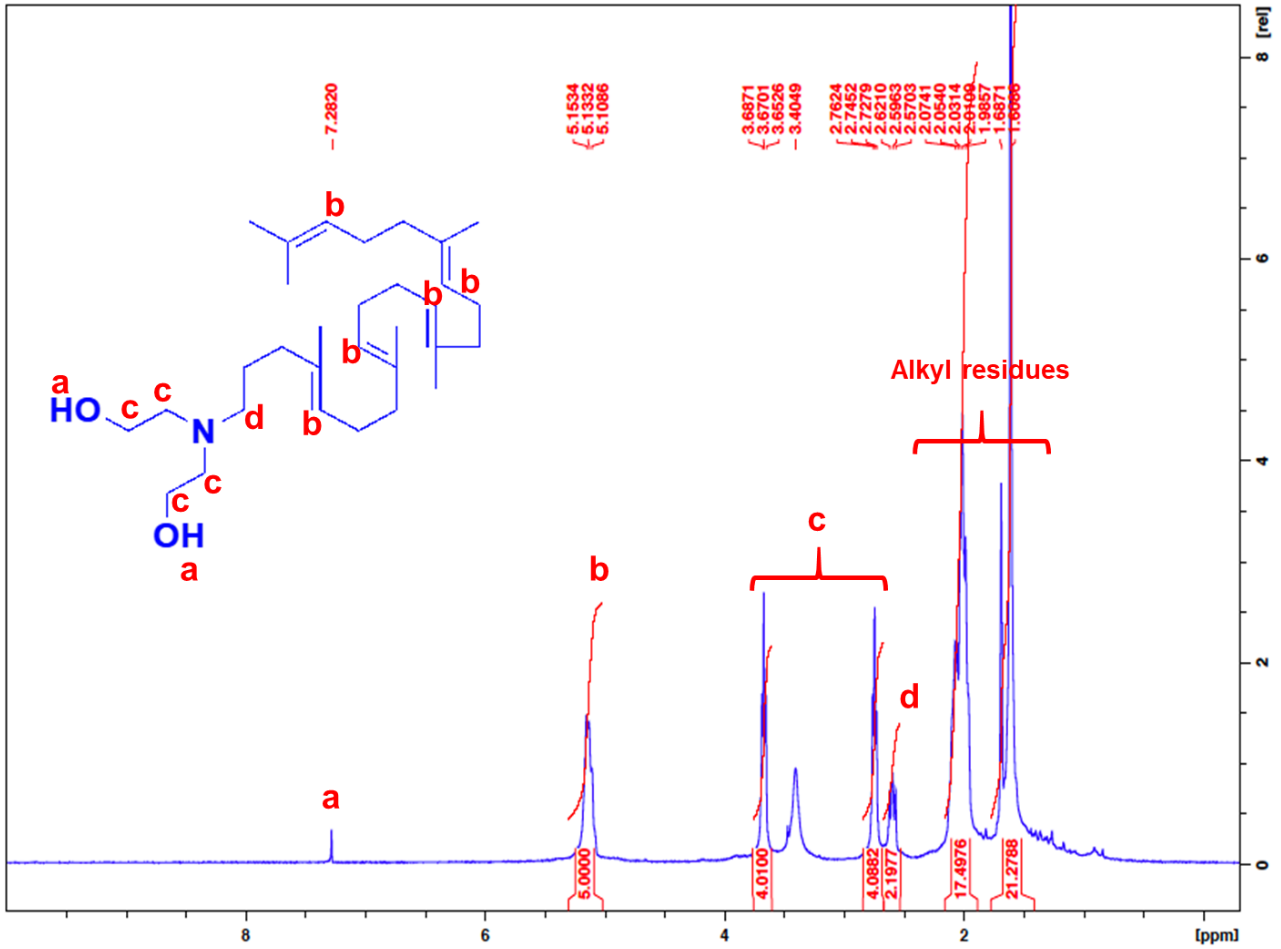

Figure S3 ^{1}H-NMR Spectrum cSq

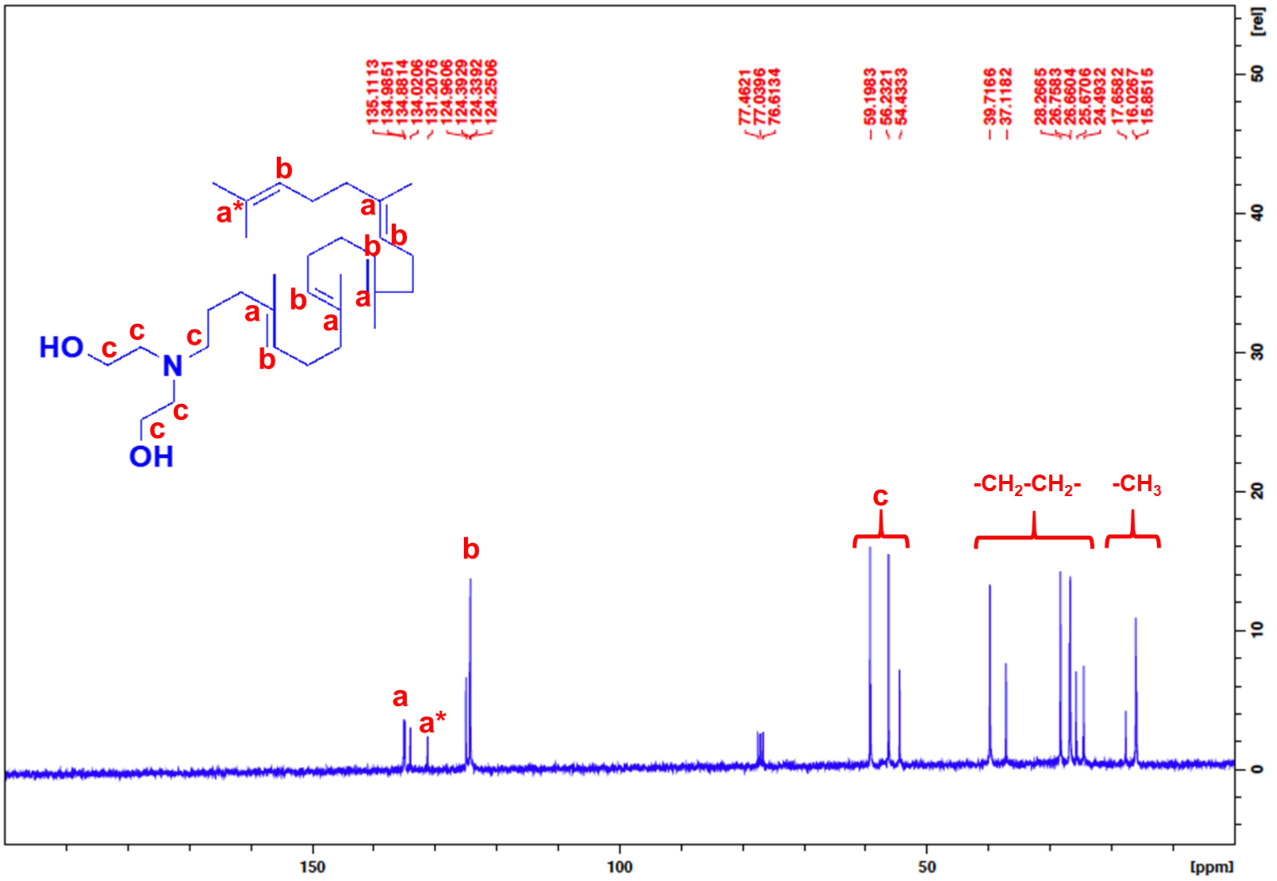

Figure S4 ^{13}C-NMR Spectrum cSq

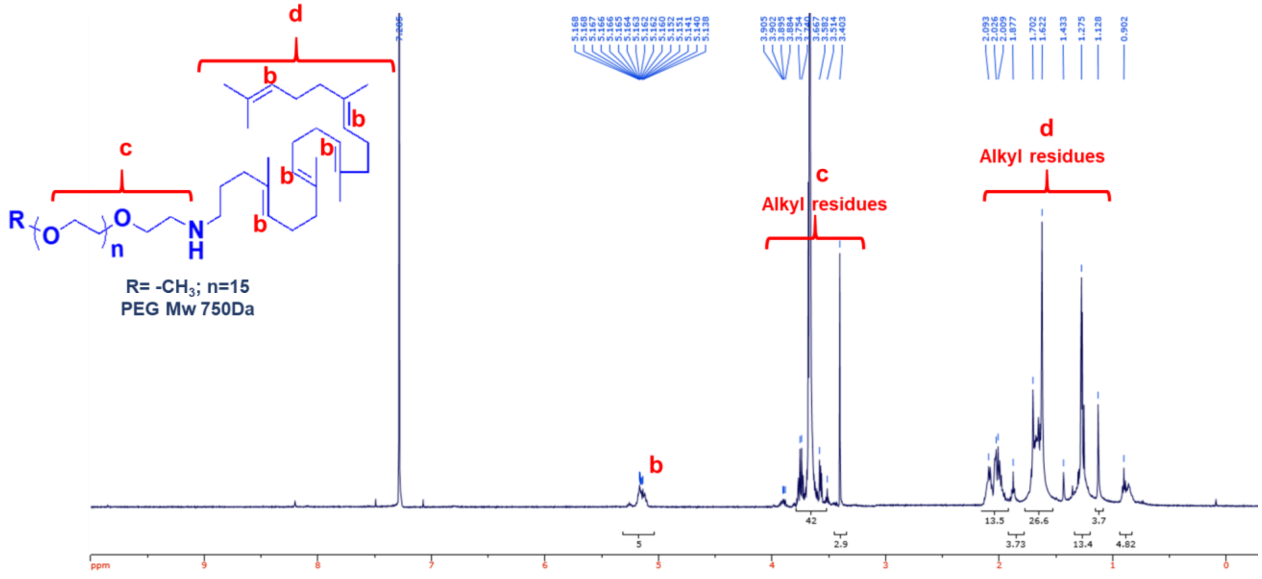

Figure S5 ^{1}H-NMR Spectrum PEG750Sq

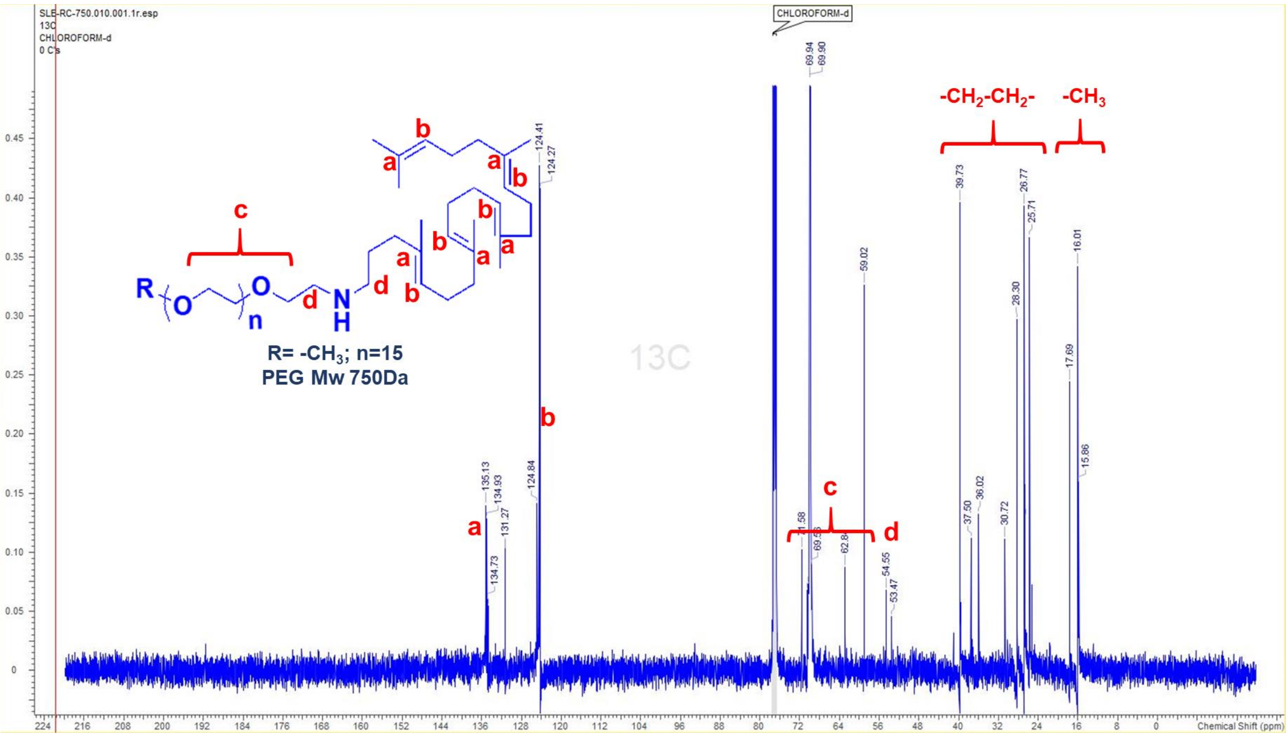

Figure S6 ^{13}C-NMR Spectrum PEG750Sq

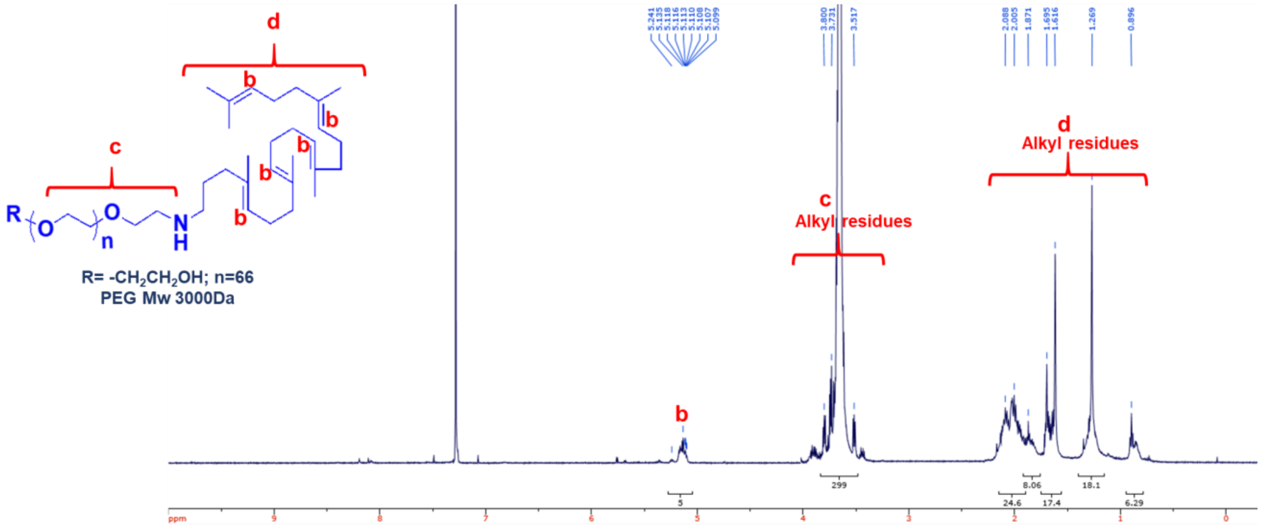

Figure S7 ^{1}H-NMR Spectrum PEG3000Sq

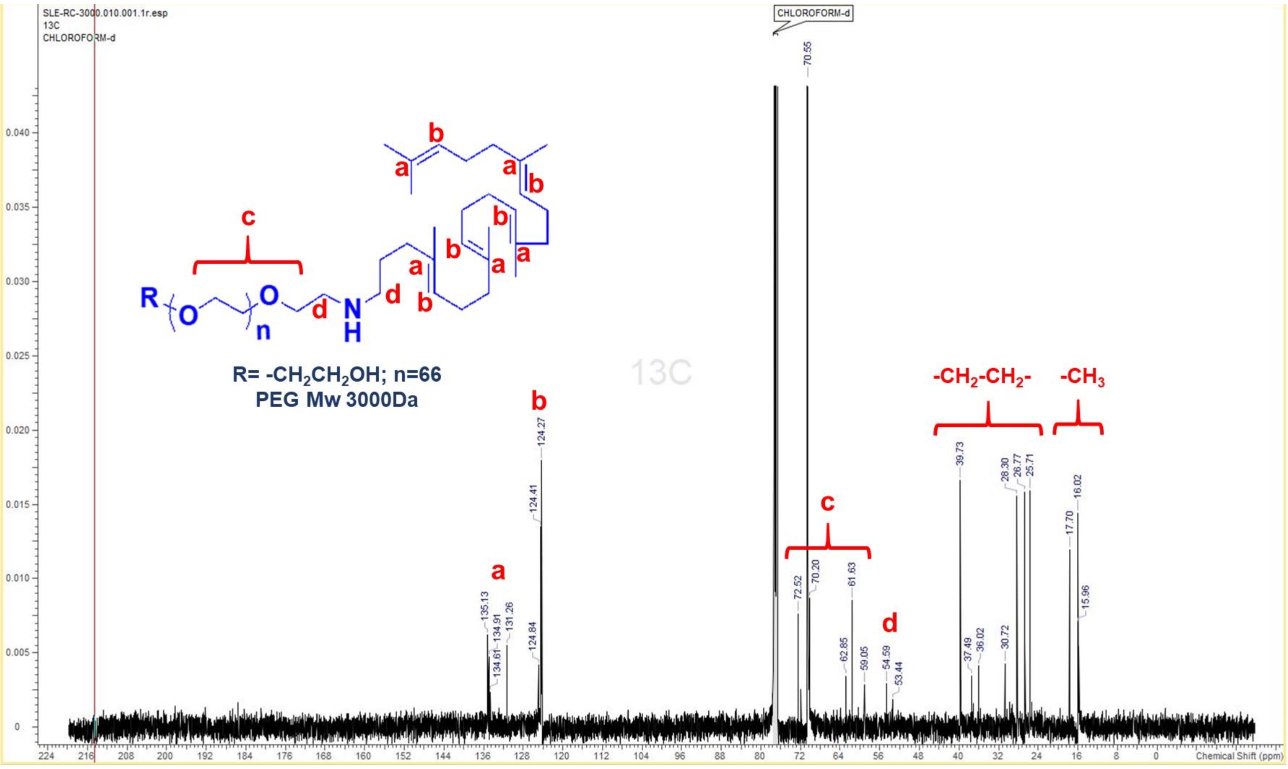

Figure S8 ^{13}C-NMR Spectrum PEG3000Sq

2 Influence of preparation factors on drug-free aSq- and cSq-NPs characteristics

Table S1 Influence of SqD-concentration in organic solvent or in water on NP characteristics measured by dynamic and electrophoretic light scattering. Results are presented as mean ± SD. n=3 for aSq and n=2 for cSq experiments, measurements in triplicates.

Squalenyl-Derivative	[mg] Sq/ mL THF	[mg] Sq/ mL H_2O	size [nm]	PdI	Zetapotential [mV]
aSq-NPs	25	1	163.6±4.4	0.113±0.004	-30.5±1.5
	10		159.5±1.7	0.107±0.026	-30.6±1.6
	7.5		134.1±0.4	0.124±0.000	-30.3±0.3
	5		111.8±0.3	0.098±0.008	-25.2±0.6
	2.5		93.1±1.5	0.147±0.004	-18.9±2.8
	25	0.5	151.5±2.2	0.112±0.022	-28.1±0.8
		1	163.6±4.4	0.113±0.004	-30.5±1.5
		2	156.8±1.7	0.087±0.011	-40.3±2.5
		5	158.5±4.0	0.104±0.051	-24.9±0.5
		10	157.3±4.5	0.099±0.017	-26.7±0.5

cSq-NPs	20	1	N.A.	N.A.	N.A.
	10		218.0±6.0	0.185±0.026	40.3±1.7
	7.5		202.9±4.9	0.140±0.012	43.4±2.0
	5		183.9±3.4	0.134±0.026	41.4±2.8
	2.5		193.4±6.1	0.109±0.015	38.0±1.6
	7.5	0.5	190.4±5.0	0.137±0.013	41.1±1.6
		1	202.9±4.9	0.140±0.012	43.4±2.0
		2	196.0±3.0	0.156±0.014	44.3±2.6

N.A. Measurements not possible due to high polydispersity

3 Protein-Sq-NPs interactions

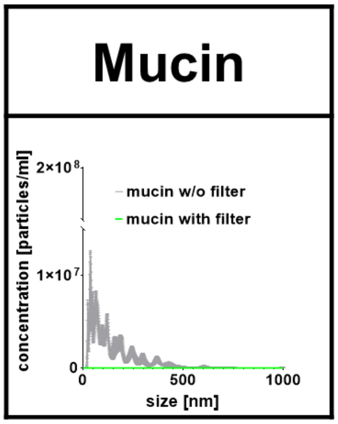

Figure S9 Mucin control for mucin glycoproteins interaction study with fluorescent SqD-NPs. Grey line: 0.1 % mucin-solution without fluorescence filter; green line: 0.1 % mucin-solution with fluorescence filter. Results are presented as mean ± SE.

Table S2 Mucin glycoproteins interaction study with fluorescent SqD-NPs. Results of particle sizes reported as mean ± SD of at least three individual experiments of number-weighted distribution of the NPs analyzed by NTA using fluorescence filter.

**SqD**	**with mucin-solution** **size [nm]** mean ± SD	**without mucin-solution** **size [nm]** mean ± SD
**aSq**	928.07±51.43	159.47±21.71
**cSq**	958.73±15.92	179.53±31.66
**PEG750Sq**	575.20±97.66	228.18±37.23

4 Confocal Laser Scanning Microscopy (CLSM) imaging of the drugs dual-loaded cSq-NPs

The ability of dual-drug loading into SqD-NPs was shown by means of CLSM images. For this purpose, cSq-NPs were loaded with the hydrophilic drug FITC-albumin (green) to the outer shell by charge interactions and nile red (red) into the core by hydrophobic interactions, resulting in a drug loading capacity of 0.5 % for each individual drug. The simultaneous presence of both model drugs was observed by CLSM (yellow). The diluted dual-loaded cSq-NPs suspension was observed using CLSM (confocal laser scanning microscope, TCS SP 8, Leica, Mannheim, Germany) equipped with a 63× water immersion objective (HC APO CS2 63×/1.20). Image analysis was done using LAS X software (Leica Application Suite X; Leica, Mannheim, Germany).

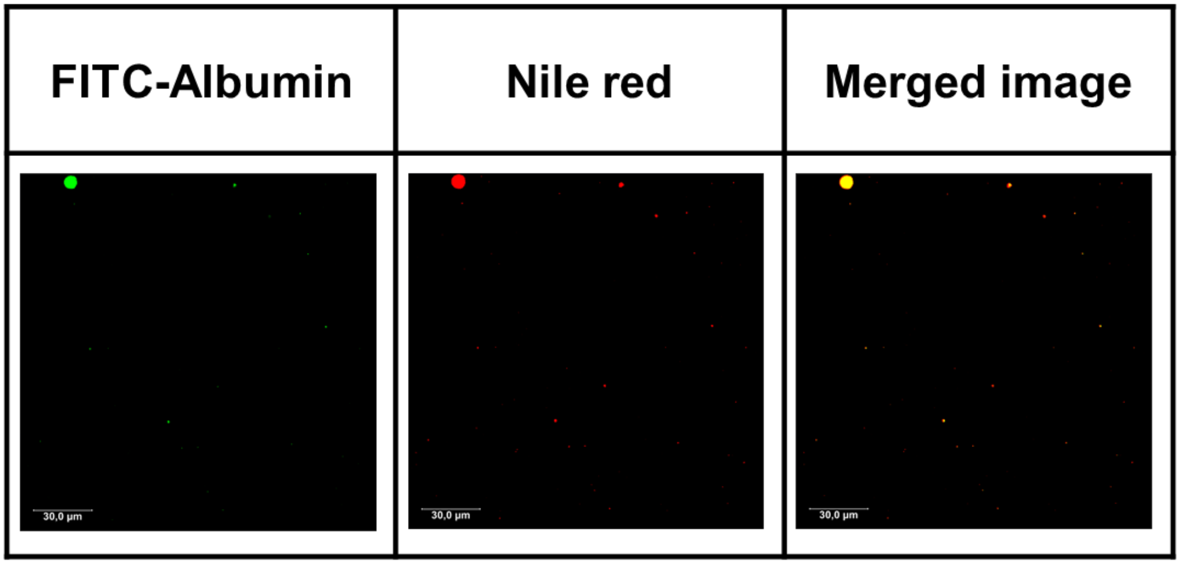

Figure S10 CLSM images of dual-loaded cSq-NPs, hydrophilic FITC-albumin (green) (0.5 % LC) on the outer shell and hydrophobic nile red (red) (0.5 % LC) in the hydrophobic core. White scale bar is 30 µm.

5 Minimum inhibitory concentration (MIC) assay

Standard microbroth dilution assays against *Staphylococcus aureus* strain Newman (*S. aureus* Newman) and *Pseudomonas aeruginosa* strain PA14 wild type (PA14) were accessed to investigate the antimicrobial properties of tigecycline loaded aSq-NPs and colistin loaded aSq-NPs while the solutions of free antibiotics and drug-free SqD-NPs prepared in water served as controls. In brief, *S. aureus* Newman and PA14 bacteria were cultured in LB Broth and PPGAS medium, respectively, which was then diluted to OD600 (absorption at 600 nm) 0.02 (relating to approximately 2 x 10^7 colony forming units (CFU)/mL) prior to the assay and distributed in 96 well-plate, 0.2 mL each well. The bacteria were incubated with the formulations for 16 h at 37 °C. Subsequently, a Tecan microplate reader Infinite M200Pro (Tecan, Crailsheim, Germany) was used to measure the absorbance at 600 nm, and the inhibitory concentrations of the formulations at which the growth of bacteria is inhibited by 90 % (IC90 values) were calculated by sigmoidal curve fitting of the absorbance values. Three independent experiments were conducted in triplicate.

Table S3 MIC assays determine IC90 values of colistin and tigecycline loaded SqD-NPs in comparison to free drug solutions against *Pseudomonas aeruginosa* PA14 and *Staphylococcus aureus* Newman.

**Samples**	**IC90 against P. aeruginosa PA14 (µg/mL)**	**IC90 against S. aureus Newman (µg/mL)**
Colistin	3.125	>64
Colistin loaded aSq-NPs	3.125[a]	>64[a]
Tigecycline	>64	0.25
Tigecycline loaded aSq-NPs	>64[a]	0.25[a]
Blank aSq-NPs	>100	>100
Blank cSq-NPs	>100	>100
PBS control	No inhibition	No inhibition
[a] drug amount		

6 Analytic methods of model compounds and drugs

Isoniazid, dexamethasone and **colistin** amounts were quantitatively analyzed by high performance liquid chromatography (HPLC) with a diode array detector (Dionex UltiMate 3000 system including LPG-3400 SD pump, WPS-3000 auto sampler, DAD3000 detector, and TCC-3000 column oven, Thermo-Fischer Scientific, Dreieich, Germany). The analysis was done by Chromeleon 7 (Chromeleon 6.80 SP2 build 9.68 for colistin) (Thermo Scientific Dionex, Dreieich, Germany), mean and standard deviation (SD) were calculated with Microsoft Excel 2016/2019 or GraphPad Prism 8.0.

The analytical method of **dexamethasone** was modified after Song et al. (Song et al. 2004), an isocratic method was used with a flow rate of 0.2 mL/min using a Syncronis C18 column (150×2.1 mm, 3 µm particle size, Thermo Scientific). The mobile phase consisted of acetonitrile and water 45:55 (*v/v*). The standard preparation and the sample dilution were done in methanol: water 45:55 (*v/v*) mixture. The method was linear over a range of 1-50 µg/mL (r-square value 0.9999). The method runtime was 8 minutes, with a dexamethasone retention time of 5 minutes detected at a wavelength of 240 nm, an injection volume of 5 µL and a column temperature of 30 °C. All samples were measured at least in triplicates.

The analytical method of **isoniazid** was as follows: an isocratic method was used with a flow rate of 0.2 mL/min using a Syncronis C18 column (150×2.1 mm, 3 µm particle size, Thermo Scientific). The mobile phase consisted of ammonium acetate buffer (pH 5.0, 10 mM) and methanol 65:35 (*v/v*). All standards and samples were diluted in mobile phase. The method was linear over a range of 1-15 µg/mL (r-square value 1.0000). The method run time was 8 minutes and the retention time of isoniazid 2.4 minutes detected at a wavelength of 260 nm, an injection volume of 10 µL and a column temperature of 40 °C. All samples were measured at least in triplicates.

Since **colistin** is a mixture, both main fractions (colistin A and B) were determined for drug quantification. The analysis method of colistin was used as described before by Yasar et al. (Yasar et al. 2018). Briefly, a gradient method with a constant flow rate of 1 mL/min and a stationary phase of LiChrospher® 100 RP-18 column (125×4 mm, 5 µm, Merck-Hitachi, Darmstadt, Germany) was used. The mobile phase consisted of acetonitrile (ACN) and 0.1 % TFA solution in water (TFA), driving a gradient of 20 % ACN, 80 % TFA to 50 % ACN, 50 % TFA within 2 minutes and holding for 1.5 minutes. All standards and samples were diluted in water. The method was linear over a range of 5-200 µg/mL (r-square value: 0.9955). The method run time was 6 minutes, the retention time of colistin A was 3.6 and of colistin B 3.9 minutes detected at a wavelength of 210 nm, an injection volume of 50 µL and a column temperature of 30 °C. All samples were measured at least in triplicates.

Cholesteryl BODIPY, nile red and **FITC-albumin** were quantitatively analyzed by fluorescence spectrometry (**tigecycline** by UV-absorbance) using a Tecan microplate reader Infinite M200Pro (Tecan, Crailsheim, Germany) as described before (Ho et al. 2018).

Nile red standards were prepared in ethanol over a concentration range of 0-10 µg/mL. The relative fluorescence units were measured using an extinction wavelength of 540 nm and an emission wavelength of 600 nm, resulting in r-square value of 0.9859. All measurements were done at room temperature, and all standards were measured five times.

Cholesteryl BODIPY standards were prepared in ethanol over a concentration range of 0-10 µg/mL. The relative fluorescence units were measured using an extinction wavelength of 495 nm and an emission wavelength of 550 nm, resulting in r-square value of 0.9898. All measurements were done at room temperature, and all standards were measured five times.

FITC-albumin standards were prepared in water over a concentration range of 0-31.25 µg/mL. The relative fluorescence units were measured using an extinction wavelength of 490 nm and an emission wavelength of 540 nm, resulting in r-square value of 0.9966. All measurements were done at room temperature, and all standards were measured five times.

Tigecycline standards were prepared in water over a concentration range of 0-200 µg/mL. The absorbance was measured at 353 nm, resulting in r-square value of 0.9977. All measurements were done at room temperature, and all standards were measured five times.

7 Bacteria and cell culture

7.1 Bacteria culture

Pseudomonas aeruginosa (PA) strain PA14 wild type (wt) and *Staphylococcus aureus* (S. aureus) strain Newman were purchased from DSMZ and ATCC, respectively, the German Collection of Microorganism and Cell Cultures GmbH (PA14 = DSMZ19882; *S. aureus* Newman = ATCC25904). PA14 was cultured in minimal proteose peptone glucose ammonium salt (PPGAS) medium, while *S. aureus* Newman was cultured in LB Broth medium. PPGAS was composed of 1 g/L NH_4Cl, 1.5 g/L KCl, 19 g/L Tris-HCl, 10 g/L peptone, 5 g/L glucose and 0.1 g/L $MgSO_4{\bullet}7\ H_2O$. The medium was adjusted to pH 7.2 ± 0.2 and sterilized before use. Agar solution was composed of 15.5 g/L LB agar, 10 g/L peptone, 5 g/L NaCl and 5 g/L yeast extract, the solution was sterilized before being plated.

7.2 Cell culture

The cell line A549 (ATCC, CCL-185), passage number between 27 and 56, was used in this work. The cell line was grown and maintained in T75 tissue culture flasks, with RPMI 1640 medium supplemented with 10 % (*v/v*) of inactivated FBS (Fetal Bovine Serum). The cells were fed every other day and trypsinized once reached about 80 % confluence.

References

Ho D-K, Frisch S, Biehl A, Terriac E, Rossi C de, Schwarzkopf K, Lautenschläger F, Loretz B, Murgia X, Lehr C-M. 2018. Farnesylated Glycol Chitosan as a Platform for Drug Delivery: Synthesis, Characterization, and Investigation of Mucus-Particle Interactions. Biomacromolecules. 19:3489–3501. eng. doi:10.1021/acs.biomac.8b00795.

Song Y-K, Park J-S, Kim J-K, Kim C-K. 2004. HPLC Determination of Dexamethasone in Human Plasma. Journal of Liquid Chromatography & Related Technologies. 27:2293–2306. doi:10.1081/JLC-200025726.

Yasar H, Ho D-K, Rossi C de, Herrmann J, Gordon S, Loretz B, Lehr C-M. 2018. Starch-Chitosan Polyplexes: A Versatile Carrier System for Anti-Infectives and Gene Delivery. Polymers (Basel). 10. eng. doi:10.3390/polym10030252.
